# Development and validation of a model to predict cognitive impairment in traumatic brain injury patients: a prospective observational study

**DOI:** 10.1016/j.eclinm.2024.103023

**Published:** 2025-01-02

**Authors:** Xiaofang Yuan, Qingrong Xu, Fengxia Du, Xiaoxia Gao, Jing Guo, Jianan Zhang, Yehuan Wu, Zhongkai Zhou, Youjia Yu, Yi Zhang

**Affiliations:** a Department of Rehabilitation Medicine, Third Affiliated Hospital of Soochow University, Changzhou, China; b Department of Anesthesiology, Third Affiliated Hospital of Soochow University, Changzhou, China; c Department of Nursing, Suzhou Xiangcheng People's Hospital, Suzhou, China; d Jiangsu University of Technology, Changzhou, China; e Department of Anesthesiology, Suzhou Xiangcheng People's Hospital, Suzhou, China; f Yangzhou University School of Medicine, China

**Keywords:** Traumatic brain injury, Early, Cognitive impairment, Prediction model

## Abstract

**Background:**

Traumatic brain injury (TBI) is a significant public health issue worldwide that affects millions of people every year. Cognitive impairment is one of the most common long-term consequences of TBI, seriously affect the quality of life. We aimed to develop and validate a predictive model for cognitive impairment in TBI patients, with the goal of early identification and support for those at risk of developing cognitive impairment at the time of hospital admission.

**Methods:**

The training cohort included 234 TBI patients, all of whom were admitted to the Department of Neurosurgery at the Third Affiliated Hospital of Soochow University from May 2017 to April 2020. These patients were selected from our previously published studies. Baseline characteristics, medical history, clinical TBI characteristics, treatment details, and vital signs during hospitalization were screened via least absolute shrinkage and selection operator (LASSO) and logistic regression to construct a predictive net risk score. The derived score represents an estimate of the risk of developing cognitive impairment in patients with TBI. A nomogram was constructed, and its accuracy and predictive performance were evaluated with the area under the receiver operating characteristic curve (AUC), calibration curves, and clinical decision curves. For the validation cohort, data were prospectively collected from TBI patients admitted to the Department of Neurosurgery at the Third Affiliated Hospital of Soochow University from March 1, 2024 to August 30, 2024, according to the inclusion and exclusion criteria. This study is registered with the Chinese Clinical Trial Registry (ChiCTR) at http://www.chictr.org.cn/ (registration number: ChiCTR2400083495).

**Findings:**

The training cohort included 234 patients. The mean (standard deviation, SD) age of the patients in the cohort was 47.74 (17.89) years, and 184 patients (78.63%) were men. The validation cohort included 84 patients with a mean (SD) age of 48.44 (14.42) years, and 68 patients (80.95%) were men. Among the 48 potential predictors, the following 6 variables were significant independent predictive factors and were included in the net risk score: age (odds ratio (OR) = 1.06, 95% confidence interval (CI): 1.03–1.08, P = 0.00), years of education (OR = 0.80, 95% CI: 0.70–0.93, P = 0.00), pulmonary infection status (OR = 4.64, 95% CI: 1.41–15.27, P = 0.01), epilepsy status (OR = 4.79, 95% CI: 1.09–21.13, P = 0.04), cerebrospinal fluid leakage status (OR = 5.57, 95% CI: 1.08–28.75, P = 0.04), and the Helsinki score (OR = 1.53, 95% CI: 1.28–1.83, P = 0.00). The AUC in the training cohort was 0.90, and the cut-off value was 0.71. The AUC in the validation cohort was 0.87, and the cut-off value was 0.63. The score was translated into an online risk calculator that is freely available to the public (https://yuanxiaofang.shinyapps.io/Predict_cognitive_impairment_in_TBI/).

**Interpretation:**

This model for predicting post-TBI cognitive impairment has potential value for facilitating early predictions by clinicians, aiding the early initiation of preventative interventions for cognitive impairment.

**Funding:**

This research was supported by Science and Technology Development Plan Project of ChangZhou (CJ20229036); Science and Technology Project of Changzhou Health Commission (QN202113).

**Research in context**

**Evidence before this study**

Post-traumatic brain injury (TBI) cognitive impairment is a complex condition influenced by multiple factors following brain injury. Several predictive factors have been identified, including the severity of the initial injury, as assessed by the Glasgow Coma Scale; the nature and location of the injury; and the duration of unconsciousness or coma. Furthermore, patient-specific factors such as age, preexisting neurological or psychiatric conditions, and genetic predispositions also play crucial roles. However, despite these insights, there has yet to be a universally accepted predictive model for post-TBI cognitive outcomes due to the variability in individual responses and recovery trajectories.

**Added value of this study**

We found that age, years of education, pulmonary infection status, epilepsy status, cerebrospinal fluid leakage, and the Helsinki score were independent predictors of cognitive impairment in patients with TBI. We built a visual online risk calculator based on these factors for the early prediction of cognitive impairment, and validation using a prospective cohort confirmed the accuracy and conformity of the model and revealed improved net benefit.

**Implications of all the available evidence**

In our study, we investigated the potential factors influencing the development of cognitive deficits after TBI. This model provides clinicians with a simple and intuitive tool that will help with the early identification of high-risk patients and the customization of intervention strategies, potentially reducing long-term cognitive deficits and improving the overall quality of life of TBI survivors.

## Introduction

Traumatic brain injury (TBI) is a series of symptoms and pathological changes caused by external force on the head, affects more than 50 million people worldwide each year, creating a major global health crisis with immediate and long-term consequences and imposing significant medical, social, and economic burdens.^1^^,^^2^ Despite improvements in emergency care and rehabilitation, the long-term impact of TBI remains severe, particularly regarding cognitive impairments.^3^ Current research has focused largely on acute TBI management and immediate physical outcomes,^4^ with few studies addressing cognitive impairment in the later stages. However, there is growing recognition of the need to address the cognitive and psychological sequelae of TBI.^5^^,^^6^ Cognitive impairments, such as deficits in memory, attention, and executive functions, present substantial challenges to rehabilitation and reintegration into daily life. The complexity and variability of TBI outcomes highlight the urgent need for comprehensive studies on factors influencing cognitive recovery.

The factors influencing cognitive deficits post-TBI are multifaceted and include the severity of the injury, the location and extent of brain damage, preexisting health conditions, age, and the time elapsed before receiving medical intervention.^7^, ^8^, ^9^ Early identification of these factors is crucial for improving the prognosis of patients with cognitive impairments following TBI. Proactive and timely interventions can mitigate the long-term effects of these impairments, promoting better recovery and functional outcomes for individuals. Preventing cognitive impairments after TBI is essential for several reasons. First, early and accurate diagnosis enables the implementation of targeted rehabilitation strategies, which can significantly enhance cognitive recovery. Second, understanding and addressing the underlying factors can help healthcare professionals develop personalized treatment plans, reducing the risk of long-term disability. Finally, preventive measures can alleviate the socioeconomic burden on families and healthcare systems by decreasing the need for prolonged medical care and support services. Although numerous studies have explored the prognosis of TBI, including survival, depression, neuropsychiatric outcomes, and return to work,^5^^,^^6^^,^^8^ there remains a notable absence of prediction models specifically designed to address cognitive impairments within the context of TBI prognosis.

Therefore, we aimed to develop and validate a predictive model for cognitive impairment in TBI patients, with the goal of early identification and support for those at risk of developing cognitive impairment at the time of hospital admission.

## Methods

### Study design and population

The Ethics Committee of the Third Affiliated Hospital of Soochow University approved this study, the Ethics Number is 2024 (Scientific) No. 030. Written informed consent was obtained from all participants. The study involved two cohorts: a training cohort and a validation cohort. The training cohort included 234 TBI patients, all of whom were admitted to the Department of Neurosurgery at the Third Affiliated Hospital of Soochow University from May 2017 to April 2020. These patients were selected from our previously published studies.^10^, ^11^, ^12^, ^13^ The validation cohort included data from TBI patients admitted to the Department of Neurosurgery at the Third Affiliated Hospital of Soochow University from March 1, 2024 to August 30, 2024. Data from these patients were systematically collected and analyzed. The inclusion criteria were patients who were aged 18 years or older, who experienced their first occurrence of cranial trauma, and who were fully conscious at discharge. The exclusion criteria included concurrent diseases causing central or peripheral nervous system damage, diseases leading to cognitive impairment, cognitive impairment impeded by consciousness disorders, severe aphasia, and refusal of follow-up and cognitive testing one month after discharge. Patients who met the inclusion criteria and were not excluded by these conditions were enrolled in the study. Systematic data collection and analysis were conducted to identify key indicators affecting post-TBI cognitive outcomes.

### Sample size calculation

We calculated the sample size on the basis of the events per variable (EPV) metric,^14^^,^^15^ a widely accepted method in statistical analyses. In our training cohort, the incidence of cognitive impairments one month post-TBI was 0.72. Given our intention to include six predictor variables and set the EPV to 10, we calculated the required sample size via the following formula:

$$\text{Sample}\;\text{Size}=\frac{\text{Number}\;\text{of}\;\text{Variables}\;\times \;\text{EPV}}{1\;-\;\text{Incidence}\;\text{Rate}}=\frac{6\;\times \;10}{1\;-\;0.72}=214$$

### Data collection

The data collected included the following: (1) baseline characteristics (sex, age, years of education, marital status, body mass index (BMI), smoking status, and alcohol consumption status); (2) medical history (history of hypertension, diabetes, stroke, and malignancy); (3) clinical TBI characteristics (initial Glasgow Coma Scale (GCS) score; TBI radiological features, including major injury sites (subdural injury, epidural injury, and intracerebral contusion); initial computed tomography (CT) scores (Marshall score and Helsinki score); presence of skull fractures; and cerebrospinal fluid (CSF) leakage status); (4) treatment details (occurrence of epilepsy, pneumonia, intracranial infections, and blood transfusions during hospitalization; surgical treatments, including brain surgery and tracheotomy; admission to the Neurological Intensive Care Unit (NICU), defined as receiving care in the NICU at any time during the hospital stay regardless of duration); and (5) vital signs during hospitalization (temperature and mean arterial pressure at admission and discharge; and hematological parameters, including platelet count, the neutrophil‒lymphocyte ratio (NLR), and hemoglobin, mean corpuscular hemoglobin concentration (MCHC), albumin, gamma‒glutamyl transferase (γ‒GT), total cholesterol, triglycerides, high-density lipoprotein, low-density lipoprotein, creatinine, potassium, blood glucose, C-reactive protein (CRP), and D-dimer levels).

### Definitions

We used the Montreal Cognitive Assessment (MoCA) to evaluate cognitive ability during the follow-up of TBI patients in the training and validation cohorts. The MoCA is a widely used tool with high sensitivity and specificity for assessing cognitive impairment, with total scores ranging from 0 to 30. Lower MoCA scores indicate poorer cognitive function.^16^ We referred to the article published by Naresh Panwar, which concluded that the cut-off value for the MoCA assessment of patients with post-TBI cognitive impairment is 22.5 points.^17^

### Data analysis

Data processing and analysis were performed via SPSS 25.0. We assessed the pattern of missing data using the md. pattern () function from the “mice” package in R to determine the missing data mechanism. We used multiple imputation to handle missing data, performing 10 imputations based on standard recommendations under the confirmed MAR mechanism. The Multiple Imputation by Chained Equations (MICE)^18^ approach was employed using the mice package in R. Continuous variables were imputed with predictive mean matching, categorical variables were imputed with logistic regression, and multinomial variables were imputed with polytomous regression (multinomial logistic regression). All relevant covariates, including predictors, the outcome variable, and other variables not included in the predictive model, were included in the imputation model to capture the relationships between variables.

Descriptive analysis involved the Shapiro–Wilk test to assess the normality of continuous data. Normally distributed continuous data are presented as the mean (standard deviation, SD), whereas nonnormally distributed data are reported as the median (interquartile range) [M (IQR)]. Categorical data are expressed as frequencies (percentages). Comparisons of normally distributed continuous data between two groups were conducted via the t test, whereas nonnormally distributed data were compared via the Mann–Whitney U test. Comparisons of categorical data between two groups were performed via the χ^2^ test or Fisher's exact test. A 2-sided P < 0.05 indicated a statistically significant association. Correlation analysis of independent variables was performed via R 4.3.3. To conduct these analyses, we used the cor () function in the “stats” package in R, which allows for the selection of either Pearson or Spearman methods depending on the data characteristics and research question. The resulting coefficients were then visualized using heatmaps, crafted with the ‘ggplot2’ and ‘corrplot’ packages in R, enabling customizable and intuitive graphical representations of the relationships.

Several algorithms of the machine learning algorithms prediction model were compared through pre-experimental research, include train support vector machine (SVMLinear, e1071 package), random forest (RF, randomForest package), K proximity (KNN, e1071 package), and general liner model (GLM). The model performance was evaluated in the training cohort and validation cohort respectively, including the differentiation evaluation including receiver operating characteristic (ROC) curve (AUC), sensitivity, specificity. Calibration curve, clinical decision (DCA) curve was used to evaluate the effectiveness. In training cohort, the AUC in SVMLinear model, RF model, KNN model, GLM model was 0.91, 0.99, 0.92, and 0.91 respectively. In validation cohort, the AUC in SVMLinear model, RF model, KNN model, GLM model was 0.81, 0.81, 0.77, and 0.81 respectively. The results showed that the traditional modeling method of our dataset is superior to the four methods of Machine learning algorithms. Appendix Figures S1–S3 provides more detailed results about the machine learning algorithm.

Variable selection was conducted via the least absolute shrinkage and selection operator (LASSO) method. Independent variables with nonzero coefficients in the LASSO regression model were selected and subsequently analyzed via multivariate logistic regression (P < 0.05) to identify potential predictive factors. During the model development phase, we assessed potential collinearity issues among the variables. All tolerance values exceeded 0.1, and the variance inflation factors (VIF) were below 10, indicating no significant collinearity. Additionally, we analyzed interaction effects and found no substantial interactions among the independent variables, with the P-values for interaction remaining above 0.1.

A nomogram was constructed to predict post-TBI cognitive impairment. The total nomogram score was the sum of the points assigned to each risk factor, with higher scores indicating a greater risk of developing cognitive impairment. The discriminatory ability of the model was assessed by calculating the area under the receiver operating characteristic (ROC) curve (AUC). The optimal threshold for our diagnostic test was determined using the maximum Youden's Index, calculated as “Sensitivity + Specificity − 1”, to ensure the best balance between sensitivity and specificity. Calibration curves were plotted via the rms package, and clinical decision curves were generated via the dcurves package to validate the accuracy of the predictive model.

For the validation cohort, we predefined an acceptable performance difference (Δ) of less than 0.05 for the AUC. The observed AUC difference between the training and validation cohorts (Δ = 0.03) suggests that the sample size is adequate for evaluating the model's generalizability and applicability in prospective validation. During the study design phase, we anticipated potential challenges associated with an insufficient sample size in the validation cohort. To address this, our prospective validation cohort is designed to be ongoing, with data collection actively continuing. If critical issues arise that necessitate a larger sample size, additional prospective samples can be incorporated as needed.

### Role of the funding source

The funders had no involvement in the writing of the manuscript or in any other aspect related to the study.

## Results

### General characteristics

In our current dataset, there are 22 missing variables, for a total of 308 missing values, which constitutes approximately 2.02% of the total data points. The missing values were distributed across multiple variables, as detailed in Table S1.

The training cohort included 234 TBI patients. Among them, 170 patients (72.65%) were identified as having cognitive impairment, 130 Male, mean age 52.26, while the remaining 64 (27.35%) were cognitively normal, 54 Male, mean age 37.75. The general characteristics of cognitive impairment and cognitively normal within the training cohort are detailed in Table S2.

**Fig. 1:**
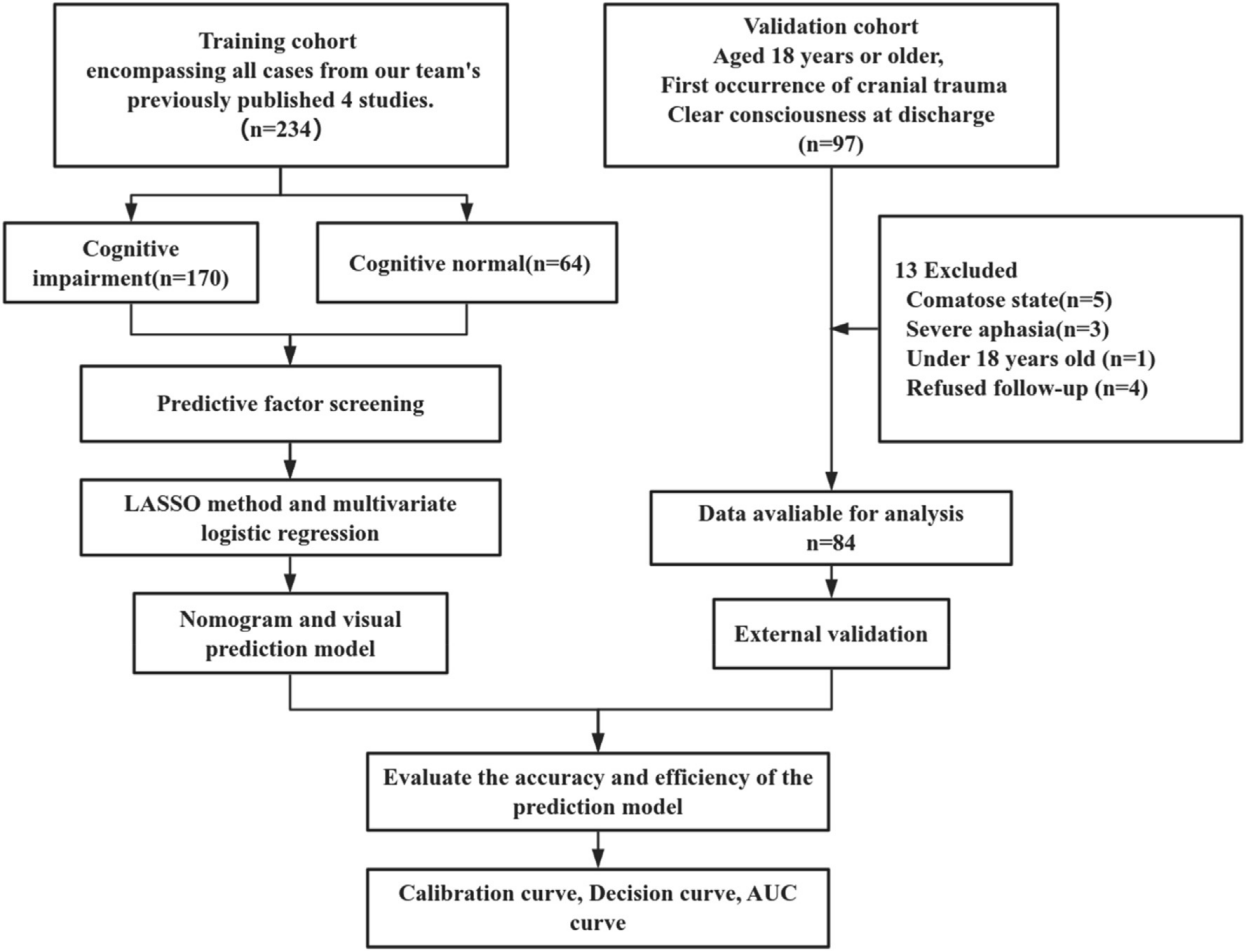
**Study flow diagram**. Our team's previously published studies (references 19, 20, 21, 22, 23). The LASSO method is the least absolute shrinkage and selection operator method. The ROC curve is the receiver operating characteristic curve. TBI refers to traumatic brain injury.

For the validation cohort, clinical information was collected for 97 patients from the Third Affiliated Hospital of Soochow University. Thirteen patients were excluded: 5 were in a comatose state, 3 had severe aphasia, 1 was less than 18 years of age, and 4 refused follow-up. Consequently, 84 patients were enrolled in the study (Fig. 1). Among them, 50 patients (59.52%) had cognitive impairment, whereas 34 (40.47%) were cognitively normal. The baseline characteristics of all patients between the training and validation cohort are presented in Table 1. Among variables in two cohorts, five variables showed significant differences between the training and validation cohorts: platelet count at discharge, serum potassium, CRP, D-dimer, and the Helsinki score.

**Table 1:** Baseline characteristics of all patients between the training and validation cohorts.^a^

Characteristics	Training cohorts	Validation cohorts	P value
	(n = 234)	(n = 84)	
Sex			0.80
Male	184 (78.63)	68 (80.95)	
Female	50 (21.37)	16 (19.05)	
Age, mean (SD), y	47.74 (17.89)	48.44 (14.42)	0.84
Years of education, median (IQR), y	9 (9–12)	9 (8–12)	0.32
Hospital stays, median (IQR), d	18 (13–29)	19 (14–28.5)	0.44
Marital status			0.21
Unmarried	48 (20.51)	12 (14.29)	
Married	184 (78.63)	72 (85.71)	
Widowed	2 (0.85)	0	
Epilepsy status			1.00
Yes	32 (13.68)	12 (14.29)	
No	202 (86.32)	72 (85.71)	
Hypertension status			1.00
Yes	63 (26.92)	21 (25.00)	
No	171 (73.08)	63 (75.00)	
Diabetes mellitus status			1.00
Yes	9 (3.85)	9 (10.71)	
No	225 (96.15)	75 (89.29)	
Malignant tumor status			1.00
Yes	3 (1.28)	0	
No	231 (98.72)	84 (100.00)	
Stroke status			1.00
Yes	5 (2.14)	0	
No	229 (97.86)	84 (100.00)	
Pulmonary infection status			0.75
Yes	59 (25.21)	13 (15.48)	
No	175 (74.79)	71 (84.52)	
Tracheotomy status			1.00
Yes	11 (4.70)	8 (9.52)	
No	223 (95.30)	76 (90.48)	
Transfusion status			1.00
Yes	68 (29.06)	25 (29.76)	
No	166 (70.94)	59 (70.24)	
NICU status			0.64
Yes	116 (49.57)	24 (28.57)	
No	118 (50.43)	60 (71.43)	
GCS score	15 (11–15)	14 (12–15)	0.27
Injuries			0.15
Epidural	40 (17.09)	20 (23.81)	
Subdural	70 (29.91)	13 (15.48)	
Intracerebral	124 (52.99)	51 (60.71)	
Brain surgery status			0.25
Yes	138 (58.97)	38 (45.24)	
No	96 (41.03)	46 (54.76)	
Intracranial infection status			1.00
Yes	3 (1.28)	3 (3.57)	
No	231 (98.72)	81 (96.43)	
Smoking status			0.48
Yes	68 (29.06)	22 (26.19)	
No	166 (70.94)	62 (73.81)	
Alcohol consumption status			0.92
Yes	41 (17.52)	13 (15.48)	
No	193 (82.48)	71 (84.52)	
Cerebrospinal fluid leakage status			0.58
Yes	26 (11.11)	25 (29.76)	
No	208 (88.89)	59 (70.24)	
Skull fracture status			0.81
Yes	110 (47.01)	51 (60.71)	
No	124 (52.99)	33 (39.29)	
Temperature on admission, median (IQR), °C	36.5 (36.00–36.80)	36.40 (36.08–36.80)	0.82
Temperature at discharge, median (IQR), °C	36.50 (36.20–36.80)	36.50 (36.10–36.80)	0.84
BMI,^b^ mean (SD)	22.92 (2.46)	22.55 (2.90)	0.17
Mean arterial pressure on admission, mean (SD), mmHg	98.40 (14.22)	99.13 (14.45)	0.80
Mean arterial pressure at discharge, mean (SD), mmHg	90.29 (10.51)	91.10 (10.54)	0.55
Hemoglobin on admission, mean (SD), g/L	128.26 (18.51)	131.29 (14.88)	0.31
MCHC on admission, mean (SD), g/L	337.26 (11.19)	336.93 (10.45)	0.81
Platelet count on admission, mean (SD),109/L	189.97 (58.00)	184.68 (55.22)	0.45
NLR^c^ on admission, mean (SD), %	10.50 (11.27)	11.76 (8.00)	0.02
Hemoglobin at discharge, mean (SD), g/L	125.29 (19.44)	125.75 (16.45)	0.57
MCHC at discharge, mean (SD), g/L	334.58 (18.06)	336.92 (11.94)	0.65
NLR^c^ at discharge, mean (SD), %	4.61 (3.68)	3.70 (1.91)	0.31
Platelet count at discharge, mean (SD),109/L	231.14 (82.30)	241.70 (76.79)	0.07
Albumin, mean (SD), g/L	36.40 (5.48)	36.78 (4.82)	0.32
γ-GT, mean (SD), U/L	37.77 (34.21)	40.48 (45.13)	0.42
Cholesterol, mean (SD), mmol/L	4.03 (0.88)	4.01 (0.80)	0.72
Triglyceride, mean (SD), mmol/L	1.57 (0.92)	1.83 (2.42)	0.62
HDL, mean (SD), mmol/L	1.20 (0.31)	1.17 (0.29)	0.38
LDL, mean (SD), mmol/L	1.95 (0.56)	2.02 (0.66)	0.71
Creatinine, mean (SD), μmol/L	83.61 (94.30)	75.17 (21.96)	0.12
Serum potassium, mean (SD), mmol/L	4.30 (0.38)	4.19 (0.55)	0.04
Blood glucose, mean (SD), mmol/L	6.13 (2.02)	6.65 (2.26)	0.06
CRP, mean (SD), mg/L	12.89 (15.00)	20.62 (25.72)	0.01
D-dimer, mean (SD), mg/L	3.55 (6.38)	6.10 (7.61)	0.00
Marshall score			0.29
1	1 (0.43)	1 (1.19)	
2	33 (14.10)	9 (10.71)	
3	52 (22.22)	24 (28.57)	
4	5 (2.14)	9 (10.71)	
5	18 (7.69)	9 (10.71)	
6	125 (53.42)	32 (38.10)	
Helsinki score, mean (SD)	3.73 (3.14)	2.90 (3.25)	0.04
MoCA score	17.69 (6.46)	20.71 (5.74)	0.00
MoCA ≤22 status			0.03
Yes	170 (72.65)	50 (59.52)	
No	64 (27.35)	34 (40.48)	

Abbreviations: NICU, neurological intensive care unit; GCS score, Glasgow coma scale score; BMI, body mass index; MCHC, mean corpuscular hemoglobin concentration; NLR, neutrophil-to-lymphocyte ratio; γ-GT, glutamyl transpeptidase; HDL, high-density lipoprotein; LDL, low-density lipoprotein; CRP, C-reactive protein. On admission means within 24 h of admission; at discharge means within 24 h before discharge.

a Data are presented as the number (percentage) of patients unless otherwise indicated.

b Body mass index is calculated as weight in kilograms divided by height in meters squared.

c The neutrophil-to-lymphocyte ratio is calculated as the neutrophil count divided by the lymphocyte count.

### Correlation heatmap of the predictor variables in the training cohort

**Fig. 2:**
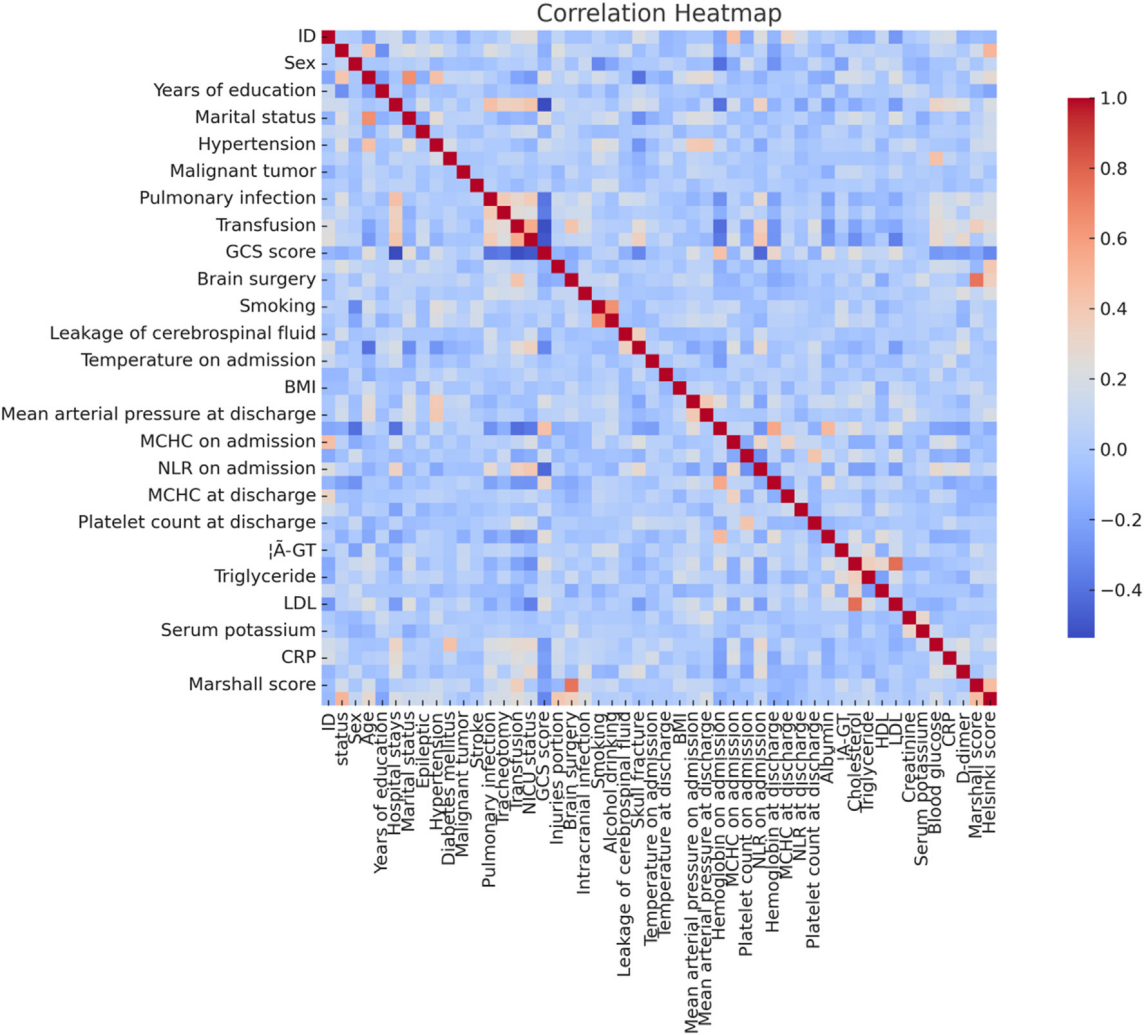
**Correlation matrix of variables in the training cohorts**.

A correlation matrix of the training cohorts is shown in Fig. 2 to illustrate the relationships between various predictor variables, and it predominantly shows weak interactions, as indicated by the light-colored cells. However, the deep blue and red areas represent strong negative and positive correlations, respectively. For example, there was a significant positive correlation between infection and hospital stay duration and a negative correlation between the GCS score and the NLR upon admission. These patterns highlight how the study variables are interconnected and influence each other, reinforcing the need to factor in these relationships during our analysis.

### Screening for predictive factors

**Fig. 3:**
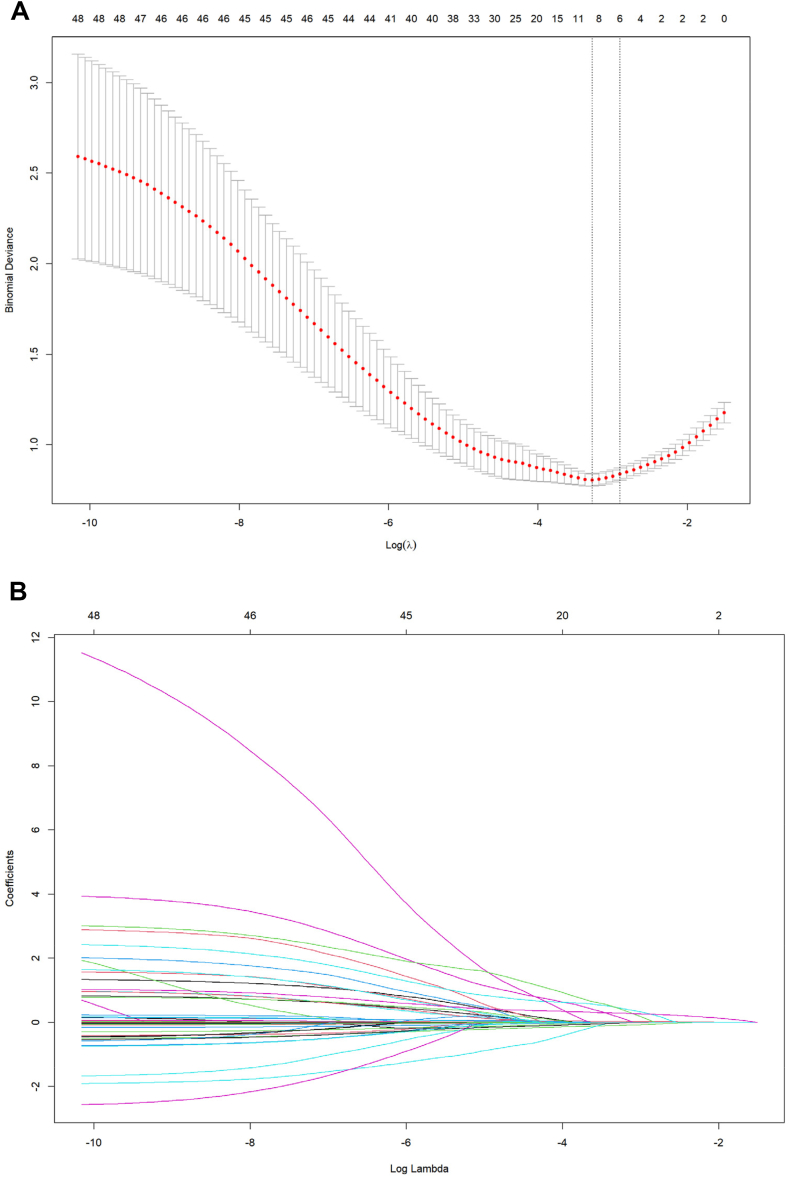
**A. Binomial deviance vs. log(λ). B. Coefficient paths for different variables**. A. Illustration of the relationship between binomial deviance and Log(λ). Each red dot represents the binomial deviance for a specific λ value, with error bars denoting the standard error of the deviance. The plot demonstrates that as Log(λ) increases, the binomial deviance initially decreases and then increases, reaching a minimum point that determines the optimal λ. Fig. 3B depicts the coefficient paths for various variables, with the x-axis representing Log(λ) and the y-axis representing the coefficients of the variables. Different colored lines denote different variables. As Log(λ) changes, the coefficients of most variables approach zero. Ultimately, eight variables with nonzero coefficients were identified, underscoring their importance in the model.

**Fig. 4:**
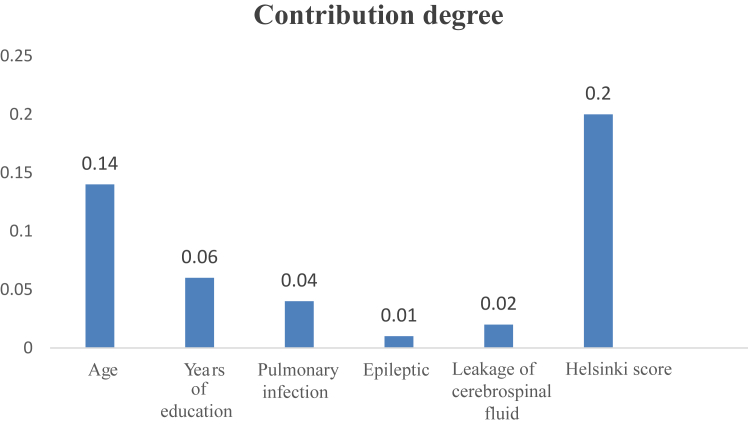
**The degree of contribution of each factor to the prediction of post-TBI cognitive impairment**.

We employed tenfold cross-validation to determine the optimal tuning parameter λ for the model and detected eight variables with nonzero coefficients: age, years of education, pulmonary infection status, hemoglobin level at discharge, epilepsy status, CSF leakage status, BMI, and the Helsinki score (Fig. 3A and B). These variables exhibited significant predictive power in the model. A multivariate logistic regression model was constructed with these significant indicators and revealed six significant risk factors for the development of post-TBI cognitive impairment: age, years of education, pulmonary infection status, epilepsy status, CSF leakage status, and the Helsinki score (Table 2). These six factors were statistically significant (P < 0.05), validating their importance in predicting post-TBI cognitive impairment. The degree of contribution of the six final predictor variables to the prediction of post-TBI cognitive impairment are shown in Fig. 4. Age had the highest contribution degree (0.14), and epilepsy status had the lowest contribution degree (0.01).

**Table 2:** Logistic regression results for risk factors associated with the development of post-TBI cognitive impairment.

Factors	Difference or OR (95% CI)	P value
Age	1.06 (1.03–1.08)	0.00
Years of education	0.80 (0.70–0.93)	0.00
Pulmonary infection	4.64 (1.41–15.27)	0.01
Epilepsy	4.79 (1.09–21.13)	0.04
Cerebrospinal fluid leakage	5.57 (1.08–28.75)	0.04
Helsinki score	1.53 (1.28–1.83)	0.00

Logistic regression results, including the odds ratios (ORs) with 95% confidence intervals (CIs) and P values for each variable. Age (OR = 1.06, 95% CI: 1.03–1.08, P = 0.00), years of education (OR = 0.80, 95% CI: 0.70–0.93, P = 0.00), pulmonary infection (OR = 4.64, 95% CI: 1.41–15.27, P = 0.01), epilepsy (OR = 4.79, 95% CI: 1.09–21.13, P = 0.04), CSF leakage (OR = 5.57, 95% CI: 1.08–28.75, P = 0.04), and the Helsinki score (OR = 1.53, 95% CI: 1.28–1.83, P = 0.000) were identified as significant predictors.

### Nomogram development and validation

**Fig. 5:**
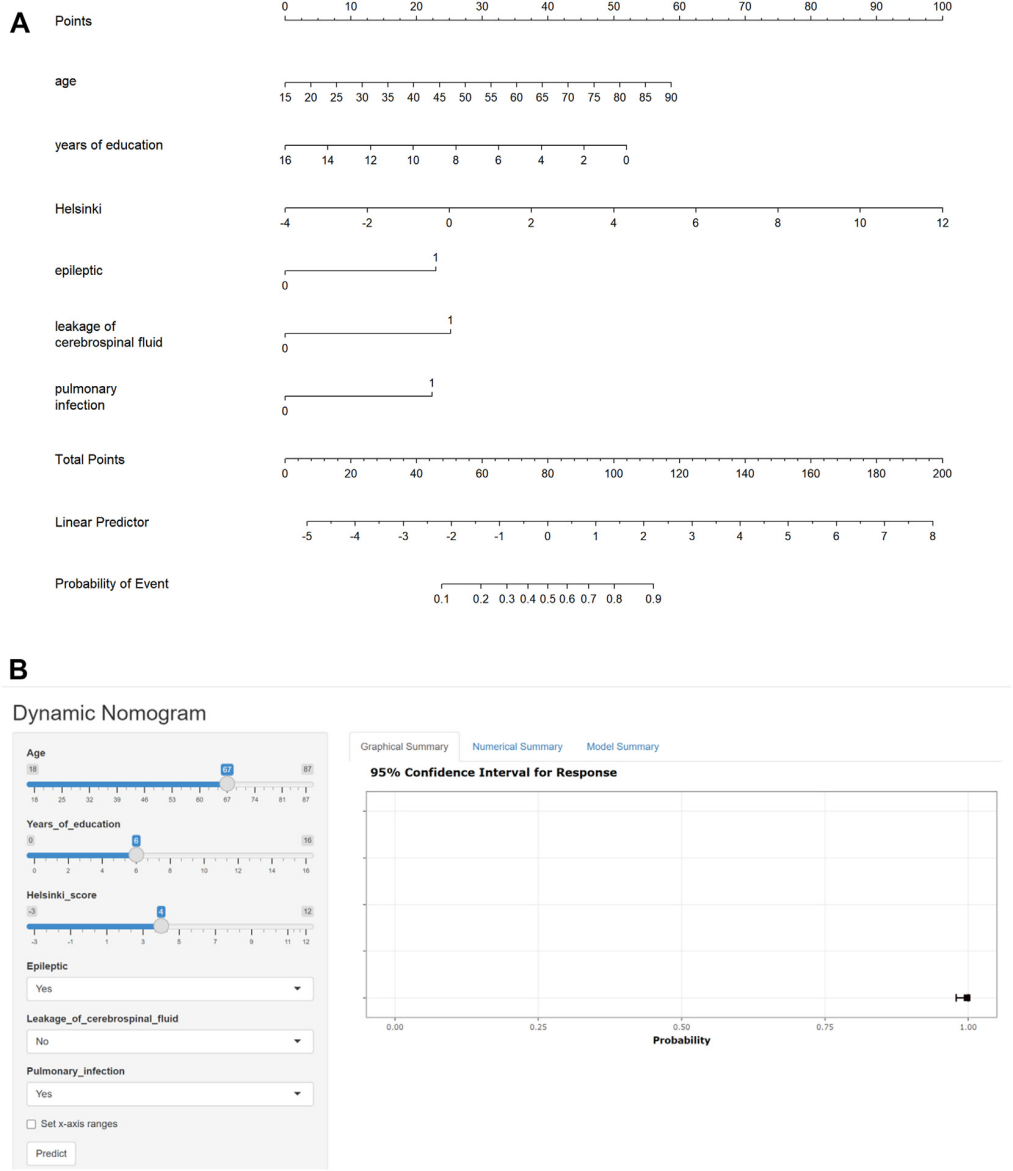
**A. Nomogram for predicting cognitive impairment in TBI patients. B. The online web-based calculator for predicting cognitive impairment in TBI patients**. A and B. The nomogram, incorporating the following predictors: age, years of education, Helsinki score, epilepsy status, CSF leakage, and pulmonary infection. Each predictor is assigned a score on a specific point scale. To use the nomogram, clinicians first locate the patient's value for each predictor on the corresponding axis. The points for each predictor are then summed to determine the total score, which is subsequently converted into a probability of developing cognitive impairment.

Using R software, we visualized the model on the basis of these six predictors and constructed a nomogram to predict post-TBI cognitive impairment (Fig. 5A). A prediction chart was created on the basis of the regression coefficients of the predictor variables (Fig. 5B). The details are available at the following website: https://yuanxiaofang.shinyapps.io/Predict_cognitive_impairment_in_TBI/. This nomogram offers a visual representation of the impact of each predictor, aiding clinicians in conducting individualized risk assessments in clinical practice. For example, the input variables include age, years of education, the Helsinki score, epilepsy status, CSF leakage status, and pulmonary infection status. On the basis of these variables, the graphical summary on the right shows the 95% confidence interval for the predicted outcome. The result indicates a probability close to 1, suggesting a high likelihood of the clinical outcome occurring given the current input conditions.

### Predictive accuracy and net benefit of the nomogram

**Fig. 6:**
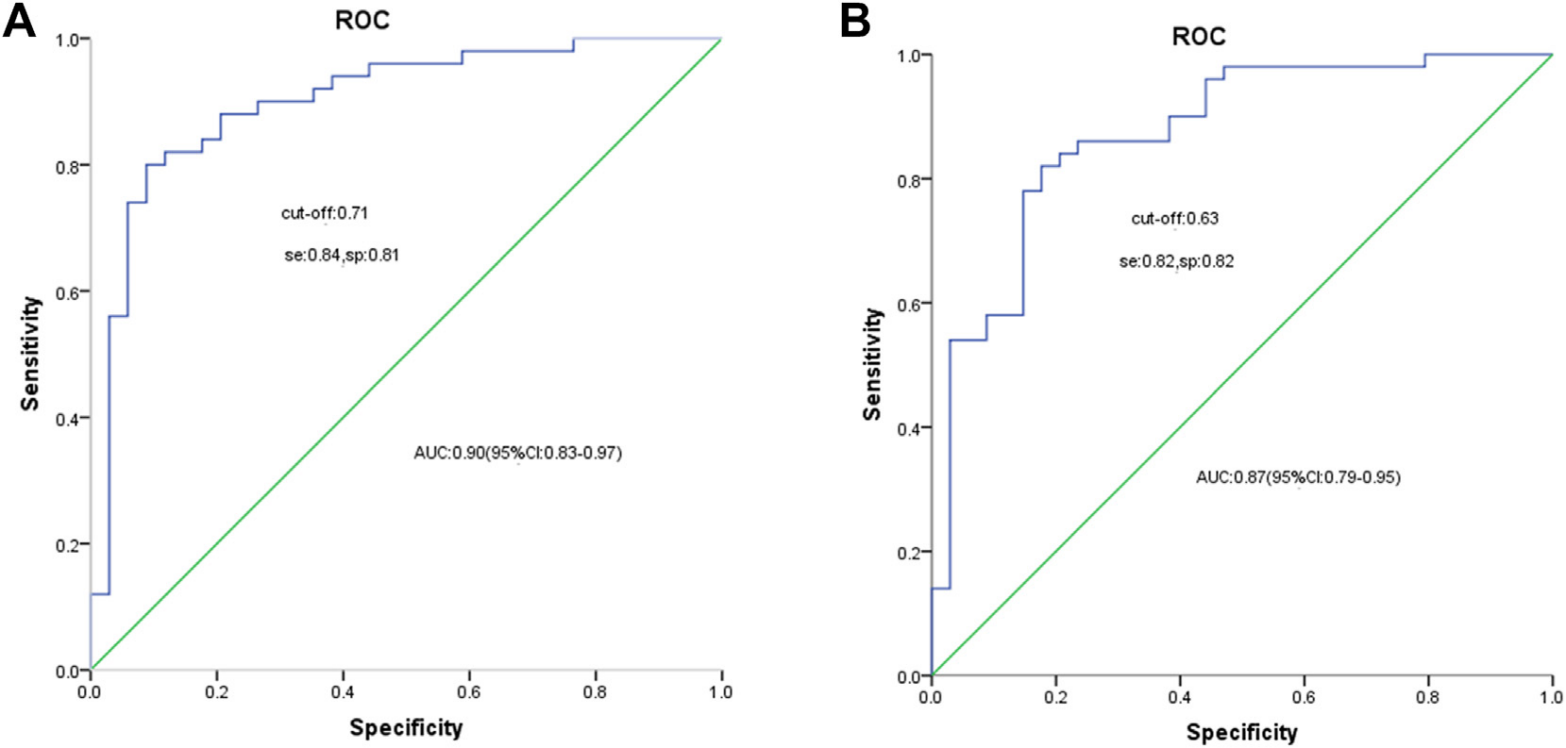
**A.** ROC curve for cognitive impairment prediction in the training cohort (AUC: 0.90, cut-off value: 0.71, sensitivity: 0.84, specificity: 0.81, Youden index: 0.65). **B.** Validation Cohort ROC Curve for Cognitive Impairment Prediction (AUC: 0.87, cut-off value: 0.63, sensitivity: 0.82, specificity: 0.82, Youden index: 0.64).

**Fig. 7:**
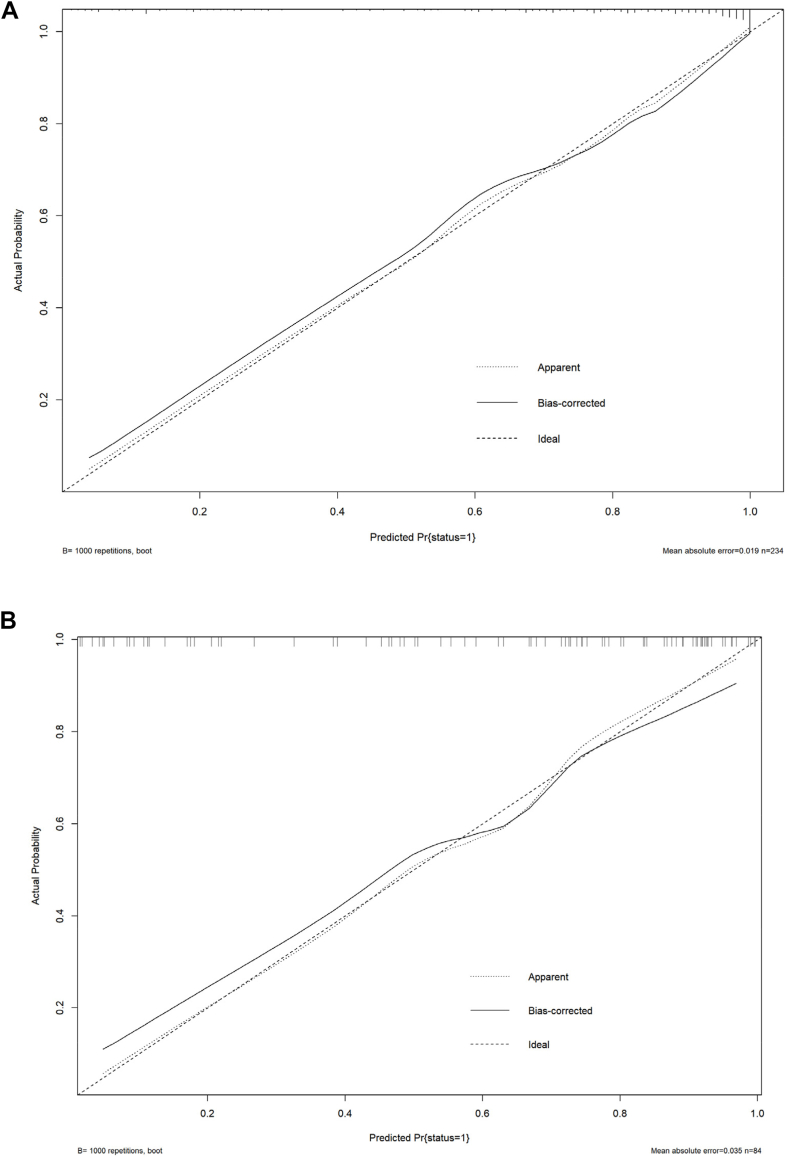
**A.** Calibration curves for predicting cognitive impairment in the training cohorts. **B.** Calibration curve for cognitive impairment prediction in the validation cohorts (Fig. 7). Decision curve analysis for the prediction of post-TBI cognitive impairment (A) Training cohort. (B) Validation cohort. Abbreviation: TBI, traumatic brain injury.

**Fig. 8:**
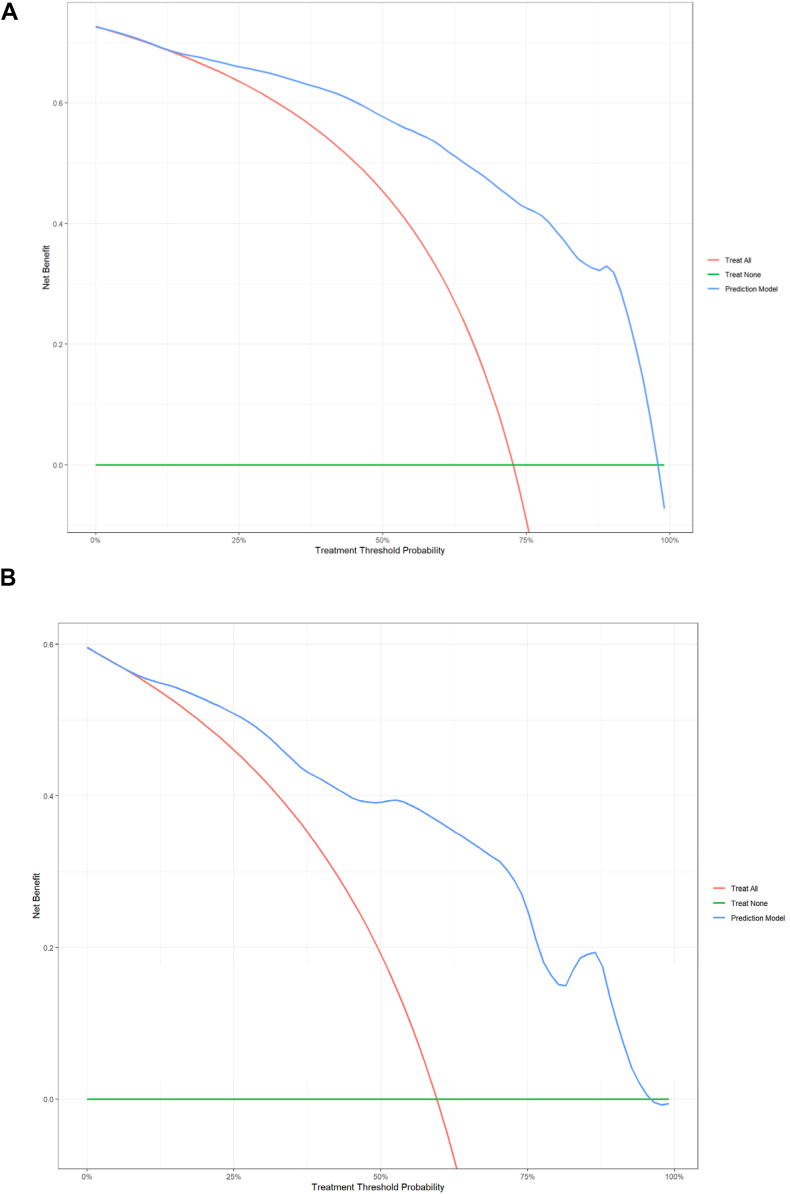
**A.** Decision curve analysis for cognitive impairment prediction in the training cohorts. **B.** Decision curve analysis for cognitive impairment prediction in the validation cohorts.

In the training cohort, the constructed nomogram exhibited excellent predictive accuracy, with an AUC of 0.91 (Fig. 6A). The calibration curve was closely aligned with the ideal diagonal line (Fig. 7A), reflecting a high level of agreement between the predicted probabilities and actual outcomes. Furthermore, decision curve analysis (DCA) of the training cohorts revealed that the model's net benefit was consistently greater than that of the two extreme strategies (including all of the variables or none of the variables) across a wide range of threshold probabilities, indicating its potential clinical utility (Fig. 8A). Net benefits for different threshold probabilities are shown in Table S3.

For validation, data from 84 patients in a prospective cohort were used to evaluate the accuracy of the model. The model achieved an AUC of 0.87 (Fig. 6B), demonstrating good predictive performance on new data. The calibration curve in the validation cohort also showed good agreement, closely following the ideal diagonal line (Fig. 7B), indicating high predictive accuracy. Additionally, the DCA in the validation cohort revealed that the model's net benefit was consistently greater than that of the two extreme strategies (treat all and treat none) across a wide range of threshold probabilities, indicating its potential clinical utility (Fig. 8B). The net benefits for different threshold probabilities are shown in Table S4.

## Discussion

In our study, we identified age, years of education, pulmonary infection status, epilepsy status, CSF leakage status, and the Helsinki score as key predictors of post-TBI cognitive impairment. On the basis of these factors, we developed an online risk calculator to predict cognitive impairment at an early stage. Validation using a prospective cohort demonstrated the model's accuracy, reliability, and superior net benefit. To our knowledge, this is the first trial to establish a prediction model specifically for cognitive impairment in TBI patients.

The incidence of cognitive impairment was 72.65% in the training cohort in our study. While this rate was used to calculate the sample size, it reflects a general level observed in the broader population. For instance, Andrew M. Bryant et al.'s^24^ research reported an incidence range of 64–79%, which is consistent with our findings. This supports the representativeness of our training cohort. Patients with brain injuries often exhibit diverse and evolving symptoms due to multiple pathophysiological mechanisms,^19^^,^^20^ such as excitotoxicity, apoptosis, and inflammation, leading to varying degrees of cognitive impairment. To detect post-TBI cognitive impairments, we used the MoCA score,^16^ which has demonstrated strong reliability and validity in evaluating brain injury cognition. The MoCA assesses various cognitive domains, including attention, calculation, memory, language, and executive function, thus providing more robust outcomes. Consistent with other Chinese studies, our study revealed that in China, TBI occurs most often in young and middle-aged males (18–65 years), with traffic accidents being the leading cause,^4^ These patients often express a strong desire to return to work, but cognitive impairment significantly hinders their ability to resume work and daily activities. Thus, early identification and management of risk factors for post-TBI cognitive impairment can reduce its incidence and alleviate the societal burden.^21^

We leveraged a diverse dataset to identify critical factors contributing to cognitive impairment in TBI patients. For the training cohort, we used retrospective data from four articles published by our team. In the validation cohort, we utilized prospective data to validate our previous findings and provide new insights into cognitive impairments following TBI. Our comprehensive analysis encompassed a wide range of independent variables. Although there were five variables showed significant differences between the training and validation cohorts, four of these variables were excluded during the feature selection process and were not included in our predictive model. The Helsinki score exhibited a slight difference between the two cohorts. Despite these differences, the AUC, calibration curve, and clinical decision curve for the training and validation cohorts remain within satisfactory ranges, supporting the robustness of our findings.

We chose the Pearson correlation as our primary method to assess linear relationships between variables because preliminary analysis indicated a linear trend between our key variables of interest. However, recognizing the potential for non-linear associations in some of our data sets, we also implemented the Spearman correlation to evaluate monotonic relationships where the assumption of normality might not hold.

The correlation heatmap revealed notable deep blue and red spots, representing strong positive or negative correlations between specific variables,^22^ including a strong positive correlation between age and hypertension, indicating that the likelihood of developing hypertension increases with age. The GCS score was negatively correlated with both hospital stay and the NLR at admission, indicating that a lower GCS score is associated with longer hospital stays. These correlations highlight the interdependence between variables, emphasizing the importance of considering these relationships in our analysis. Using advanced statistical techniques such as LASSO regression and logistic regression, we systematically identified the most influential predictors of cognitive impairment. The application of LASSO regression enabled us to efficiently identify the most relevant predictors from a comprehensive set of variables. This method ensures model parsimony and robustness, effectively avoiding overfitting while maintaining high predictive accuracy. The logistic regression analysis confirmed a more refined set of predictors, confirming their independent and significant contributions to cognitive outcomes.^23^^,^^25^

According to their degree of contribution, these predictors include age, years of education, pulmonary infection status, epilepsy status, CSF leakage status, and the Helsinki score, which offer a nuanced understanding of the multifactorial nature of post-TBI cognitive outcomes. Age is a significant factor affecting cognitive impairment. Elderly patients with TBI, despite having higher initial GCS scores, still exhibit higher overall mortality and disability rates.^26^ In general, increasing age is correlated with increased risk and severity of cognitive impairment.^4^ It is likely that older patients, owing to lower baseline cognitive function, are less able to withstand the additional burden of post-TBI cognitive impairment.

Education is another key factor influencing cognitive outcomes after TBI. Research indicates that higher levels of education in TBI patients can delay or mitigate cognitive decline by enhancing both verbal and nonverbal task performance^27^ and increasing cognitive reserve, which helps preserve brain function despite damage.^28^ Patients with more education have fewer symptoms and are more likely to achieve full recovery,^29^ with education serving as an independent predictor of disability-free recovery within one year.^30^ These findings are supported by studies demonstrating the protective effect of higher education in reducing the cognitive impacts of TBI.^27^^,^^31^

We found that the occurrence of epileptic seizures and CSF leakage are independent risk factors for post-TBI cognitive impairment. These factors have received limited attention in the context of TBI, highlighting both the innovation and significance of our model.^31^ Post-TBI epilepsy (PTE) refers to epilepsy characterized by unprovoked seizures that develop more than a week after the occurrence of TBI.^32^ A study involving 1100 individuals with TBI revealed that those with PTE exhibited poorer cognitive performance than those without PTE.^33^ In a preclinical study, compared with those with only TBI, mice with TBI followed by induced seizures demonstrated reduced learning and memory abilities in the Barnes maze test.^34^ Further research indicated that post-TBI patients with chronic seizures scored lower on cognitive assessments than those with earlier seizures during six months of inpatient rehabilitation.^35^ These findings suggest the potential benefits of antiepileptic therapy in delaying cognitive recovery in patients with early-onset epilepsy following TBI.

Along with epileptic seizures, CSF leakage may also contribute to post-TBI cognitive impairment. Meningeal rupture leading to CSF leakage results in a pathological decrease in CSF pressure. Reports of cognitive dysfunction caused by CSF leakage are rare. Gharehbagh et al. reported a case where a patient with spontaneous dural leaks exhibited cognitive dysfunction, with neuropsychological consultation revealing difficulties in concentration, memory, and fine motor skills.^36^ Schievink et al. identified behavioral variant frontotemporal dementia as a serious complication of spontaneous intracranial hypotension.^37^ These findings indicate that CSF leakage, which leads to low intracranial pressure, is associated with the development of cognitive impairment. Although reports are limited, the evidence underscores the potential cognitive risks associated with sustained low intracranial pressure.

Hospitalized TBI patients commonly develop various infections, which are correlated with injury severity and pose significant risks to recovery.^38^ In fact, lung pathology is frequently observed among TBI patients with infections, with pneumonia occurring in 30–50% of those who develop nosocomial infections.^39^^,^^40^ This is consistent with our findings and further supports the critical link between lung health and neurological outcomes. Bacterial lung infections following experimental TBI have been shown to exacerbate both neuroinflammation and neurological dysfunction. One study demonstrated that lung infection, regardless of timing, increased mortality, neuroinflammation, and motor dysfunction.^41^ Given the role of neuroinflammation in the progression of cognitive deficits, these findings underscore the need for effective management of lung infections in TBI patients to reduce the risk of further cognitive decline.

TBI classification via the GCS score ranges from mild to severe, but the GCS score can be inaccurate because of factors such as alcohol intoxication, sedation, and interrater variability.^42^ CT imaging offers a more objective alternative, providing crucial diagnostic and prognostic data on intracranial injuries.^43^ The Helsinki score, which integrates aspects of the Marshall and Rotterdam scores, is more sensitive in predicting outcomes and shows an inverse correlation with post-TBI cognitive function; higher Helsinki scores predict poorer cognitive outcomes.^44^^,^^45^ In this study, we observed a strong positive correlation between the Helsinki score and the Marshall score. Importantly, higher Helsinki scores were consistently associated with a greater incidence of cognitive impairment, underscoring the predictive value of the Helsinki score for post-TBI cognitive outcomes.

The nomogram developed in this study offers a practical tool for improving clinical decision-making in TBI care. Through the integration of six key predictors—age, years of education, pulmonary infection status, epilepsy status, CSF leakage status, and the Helsinki score. The nomogram can be used by clinicians for more precise, individualized risk assessment. The application of machine learning is rapidly increasing in this field of study, include Train support vector machine, Random Forest, K proximity and General liner models. The modeling method in this study has an AUC value of 0.90 in the training cohorts, which is similar to the results of the GLM model. An AUC value of 0.87 in the verification cohorts, which is superior to the results of the GLM model. Moreover, the AUC in this study is higher and performs better than that of the GLM model in the verification Cohort. Because of the type of predictor we used, continuous and categorical (operator-dependent) variables were included (i.e., clinical scales, radiological indicators), and the sample size of our study was small, the machine learning algorithms may not perform better than traditional regression models in predicting the outcome in TBI patients.^46^

In our study, the model performed exceptionally well with an AUC of 0.90 in the training cohort, and it maintained strong predictive accuracy in the validation cohort, with an AUC of 0.87. To evaluate the model's performance in the validation cohort using the training cohort's cut-off value, we applied the cut-off value of 0.71, determined from the training cohort, directly to the predicted probabilities of the validation dataset, it has a sensitivity of 0.74, a specificity of 0.85, and a Youden index of 0.59. Compared to the performance metrics calculated using the validation cohort's optimal cut-off value, both the sensitivity and Youden index have decreased, while the specificity has increased. The optimal Youden index in the training cohort is 0.65, while the maximum Youden index in the validation cohort is 0.64, which is very close to 0.65. Using this Youden index (0.64) in the validation cohort, we recalculated the sensitivity, specificity, and cut-off value. The results show that the sensitivity is 0.82, the specificity is 0.82, and the cut-off value is 0.63, which are consistent with the performance metrics derived from the validation cohort's optimal Youden index.

The close alignment between the predicted and actual outcomes, as demonstrated by the calibration curves, underscores the model's reliability. Additionally, DCA revealed that the nomogram consistently delivered greater clinical benefit than did universal treatment or no treatment across a wide range of thresholds, reinforcing its practical value. This tool not only improves the ability to predict cognitive impairment in TBI patients but also provides a foundation for more personalized, targeted interventions. This model could serve as a springboard for future research, including the exploration of additional predictors or the refining of the capabilities of the nomogram, with the ultimate goal of improving cognitive recovery in neurotrauma patients.

This study has several limitations that warrant mention. First, although we precisely calculated the required sample size and retrospectively collected a diverse range of predictive variables, the absence of a prospective multicentre large-sample design limits the generalizability and external applicability of our findings to some extent. Future external validation with large-sample multicentre studies is necessary to confirm the model's generalizability and broader applicability. Second, the inclusion of many mild-to-moderate TBI cases in the study may have affected the model's usefulness for detecting more severe cases. However, the high incidence of mild-to-moderate TBI in China reflects the current state of brain injuries in the country.^2^^,^^47^ Third, we categorized brain injury locations into three general types, epidural, subdural, and intracerebral, without distinguishing specific brain lobes. However, to address this limitation, we incorporated CT scoring to improve the classification of injury locations.

In this study, we found that age, years of education, the Helsinki score, epilepsy status, CSF leakage status, and pulmonary infection status were predictors of post-TBI cognitive impairment. On the basis of these perinatal predictors, we built a visual and personalized online risk calculator for post-TBI cognitive impairment, and our validation using a prospective cohort confirmed that this model was good. For each patient, higher total points reflected a greater risk of developing cognitive impairment. The visual, personalized predictor model provides clinicians with a simple and intuitive tool for the early detection and identification of post-TBI cognitive impairment, which may be important for reducing the high medical costs and long-term poor prognosis associated with post-TBI cognitive impairment.

## Contributors

Dr Yi Zhang and Yu had accessed and verified all the data in the study and take responsibility for the integrity of the data and the accuracy of the data analysis. Dr Yuan, Xu, and Du contributed equally to this study. Concept and design: Yi Zhang, Yu, and Yuan. Acquisition, analysis, or interpretation of data: Yi Zhang, Yu, Yuan, Xu, and Du. Drafting of the manuscript: Yuan, Xu, and Du. Critical revision of the manuscript for important intellectual content: Yi Zhang and Yu. Statistical analysis: Yuan, Gao, and Xu. Supervision: Yuan, Xu, Guo, and Zhou. All authors read and approved the final version of the manuscript.

## Data sharing statement

The data underlying this article will be shared on reasonable request to the corresponding author.

## Declaration of interests

None of the authors have conflicts of interest to disclose. None of the authors have financial relationships relevant to this article to disclose.

## Appendix A Supplementary data

Sample of Informed Consent Form
