# Supplementary material for: Development and validation of a model to predict cognitive impairment in traumatic brain injury patients: a prospective observational study
Source: eClinicalMedicine. 2025 Jan 2;80:103023. doi: 10.1016/j.eclinm.2024.103023 (PMC11753911; doi:10.1016/j.eclinm.2024.103023)
Supplement: Sample of Informed Consent Form [file mmc1.pdf]

临床研究受试者知情同意书

请您阅读以下材料，如果您愿意参加此项临床研究，您将了解此临床研究的性质以及如何参与其中。签署知情同意书将表明您已了解此临床研究并同意参加。按照我院医学伦理委员会要求在参与临床研究前签署知情同意书，这样可以保证您在了解临床研究的性质和参与的风险后再决定是否参与此临床研究。

1、临床研究概述及目的

您被邀请参加我院的科研课题“颅脑外伤患者认知障碍预测模型的构建和验证”。研究目的：我们旨在开发并验证脑外伤患者认知功能障碍的预测模型，目的是在入院时早期识别和支持那些有发生认知功能障碍风险的患者。

2、参加人员

纳入标准：①年龄≥18 岁；②首次发生颅脑外伤；③出院时意识完全清醒；

排除标准：伴有中枢或周围神经系统损害的疾病、导致认知障碍的疾病、意识障碍阻碍的认知障碍、严重失语、出院后 1 个月拒绝随访和认知测试。

3、方法

本课题主要研究（分组、样本量、主要观察、干预和评价等）验证队列包括 2024 年 3 月 1 日至 2024 年 8 月 30 日苏州大学附属第三医院神经外科收治的 TBI 患者的数据。系统地收集和分析了这些患者的数据。收集的数据包括：(1)基线特征（性别、年龄、受教育年限、婚姻状况、体质指数（BMI）、吸烟状况和饮酒状况）；(2)病史（高血压、糖尿病、卒中、恶性肿瘤病史）；(3)颅脑损伤临床特征(初始格拉斯哥昏迷量表（GCS）评分；TBI 影像学特征，包括主要损伤部位（硬膜下损伤、硬膜外损伤和脑内挫裂伤）；初始计算机断层扫描（CT）评分（Marshall 评分和赫尔辛基评分）；存在颅骨骨折；以及脑脊液漏情况)；(4)治疗情况(住院期间是否发生癫痫、肺炎、颅内感染、输血等情况；外科治疗，包括脑外科手术和气管切开术；进入神经重症监护病房（NICU），定义为在住院期间的任何时间接受 NICU 治疗，不论持续时间)；(5)住院期间生命体征情况(入院时、出院时体温、平均动脉压；血液学参数，包括血小板计数、中性粒细胞-淋巴细胞比值（NLR）、血红蛋白、平均红细胞血红蛋白浓度（MCHC）、白蛋白、γ -谷氨酰转移酶（γ-GT）、总胆固醇、甘油三酯、高密度脂蛋白、低密度脂蛋白、肌酐、钾、血糖、c 反应蛋白（CRP）和 d -二聚体水平)。

4、受益与风险

本项临床研究的方案经过我院医学伦理委员会审核，最大程度保护受试者的权益，并且具有科学性。研究者会全程密切观察受试者的状况，一旦发生不良事件或严重不良事件能及时采取措施，正确处理相应情况。

5、自愿参加

您可以选择不参与此项临床研究，亦可以随时退出试验，而由医生采取其它设备、药物进行治疗。

6、保密

所有在试验中收集到的您的信息都将根据法律规定的程度进行保密。在研究记录中，您的个人信息在没有您的书面许可的情况下是不会公布的。但是您的记录有可能被研究主办者，伦理委员会以及相关管理机构审查。此项试验的研究成果将以论文的形式在医学期刊上发表，不过您的个人信息在任何刊物上都将是保密的。

7、费用与补偿

此研究不会增加您的任何医疗费用。一旦您因参与这项研究而受到与该研究相关的伤害，将会得到免费治疗，由此产生的医疗费用由临床研究实施者承担。

8、研究者

负责此项临床研究的研究人员是我院具有多年临床经验的医疗技术人员，能很好的保障受试者的安全，一旦发生不良事件能及时处理，最大程度保障受试者权益。

9．权利

参加此项临床研究是自愿的。您可以选择不参加，或者您参加后可以随时退出。您的决定不会影响您目前或将来接受的治疗或其他服务。如果临床医生认为您继续参加将会严重影响您的健康，他/她可能随时决定让您退出。

有关临床研究的一般问题，请随时联系临床医生。

10、受试者声明

我已经仔细阅读上述关于临床研究的内容。我的提问均已得到满意回答。此表由我自愿签署，表明我参加此项研究的愿望。签署此同意书不能免除我的合法权利。如果有疑问，或受到与研究有关的伤害，我会与我的[redacted]床医生联系。

受试者签名：[redacted]

日　　期：2024.4.28　　**联系电话：**[redacted]

11、临床医生声明

我已经仔细的向受试者介绍了以上各项的情况。因此我确保我已用我个人所学的知识向

受试者清楚地解释了临床研究的性质、要求、风险以及他/她签名的合法性。任何医学、语言或者教育的障碍都不会妨碍志愿者理解这些问题。

医生签名：袁晓芳　　联系电话：13915847276

日　　期：2024.4.28

如果您有与自身权益相关的任何问题，或者您想反映参与本研究过程中的不满和忧虑，请联系研究者：袁晓芳，电话：13915847276；伦理委员会办公室，联系电话：0519-68870261
