# Supplementary material for: Development and validation of a model to predict cognitive impairment in traumatic brain injury patients: a prospective observational study
Source: eClinicalMedicine. 2025 Jan 2;80:103023. doi: 10.1016/j.eclinm.2024.103023 (PMC11753911; doi:10.1016/j.eclinm.2024.103023)
Supplement: Tables and Figures [file mmc2.doc]

| **Table S1. The Distribution of Missing Values Across Multiple Variables in the Training Cohort** | | | |
| --- | --- | --- | --- |
| Variable | Total Entries | Missing Values | Missing Percentage |
| Marital status | 15264 | 25 | 0.16% |
| Hypertension status | 15264 | 11 | 0.07% |
| Diabetes mellitus status | 15264 | 9 | 0.06% |
| Malignant tumour status | 15264 | 10 | 0.07% |
| Stroke status | 15264 | 6 | 0.06% |
| Smoking status | 15264 | 23 | 0.15% |
| Alcohol consumption status | 15264 | 24 | 0.16% |
| BMI | 15264 | 22 | 0.14% |
| Hemoglobin on admission | 15264 | 11 | 0.07% |
| MCHC on admission | 15264 | 11 | 0.07% |
| Platelet count on admission | 15264 | 11 | 0.07% |
| NLR on admission | 15264 | 11 | 0.07% |
| NLR at discharge | 15264 | 12 | 0.08% |
| Hemoglobin at discharge | 15264 | 12 | 0.08% |
| MCHC at discharge | 15264 | 12 | 0.08% |
| Platelet count at discharge | 15264 | 12 | 0.08% |
| Cholesterol | 15264 | 13 | 0.09% |
| Triglyceride | 15264 | 13 | 0.09% |
| HDL | 15264 | 13 | 0.09% |
| LDL | 15264 | 13 | 0.09% |
| CRP | 15264 | 16 | 0.11% |
| D-dimer | 15264 | 18 | 0.12% |
| Total | 15264 | 308 | 2.02% |

| **Table S2. General characteristics of Cognitive impairment and Cognitively normal within the Training Cohort^a^** | | | |
| --- | --- | --- | --- |
| Characteristics | Cognitive impairment | Cognitively normal | *P* value |
|  | (n =170) | (n = 64) |  |
| Sex |  |  | 1.00 |
| Male | 130 (76.47) | 54 (84.38) |  |
| Female | 40 (23.53) | 10 (15.63) |  |
| Age, mean (SD), y | 52.26 (16.19) | 35.75 (16.73) | 0.00 |
| Years of education, median (IQR), y | 9.0 (6.5-12.0) | 10.5 (9.0-14.0) | 0.00 |
| Hospital stays, median (IQR), d | 18.5 (13.0-32.0) | 17.0 (11.75-24.0) | 0.03 |
| Marital status |  |  | 0.71 |
| unmarried | 142 (85.53) | 22 (34.37) |  |
| married | 26 (15.29) | 42 (65.63) |  |
| widowed | 2 (1.18) | 0 |  |
| Epilepsy status |  |  | 1.00 |
| yes | 28 (16.47) | 4 (6.25) |  |
| no | 142 (85.53) | 60 (93.75) |  |
| Hypertension status |  |  | 1.00 |
| yes | 56 (32.94) | 7 (10.94) |  |
| no | 114 (67.06) | 57 (89.06) |  |
| Diabetes mellitus status |  |  | 1.00 |
| yes | 8 (4.71) | 1 (1.56) |  |
| no | 162 (95.29) | 63 (98.44) |  |
| Malignant tumour status |  |  | 0.82 |
| yes | 2 (1.18) | 1 (1.56) |  |
| no | 168 (98.82) | 63 (98.44) |  |
| Stroke status |  |  | 0.71 |
| yes | 4 (2.35) | 1 (1.56) |  |
| no | 166 (97.65) | 63 (98.44) |  |
| Pulmonary infection status |  |  | 0.00 |
| yes | 31 (31.18) | 6 (9.38) |  |
| no | 117 (68.82) | 58 (90.63) |  |
| Tracheotomy status |  |  | 0.04 |
| yes | 11 (6.47) | 0 (0.00) |  |
| no | 159 (93.53) | 64 (100.00) |  |
| Transfusion status |  |  | 0.40 |
| yes | 52 (30.59) | 16 (25.00) |  |
| no | 118 (69.41) | 48 (75.00) |  |
| NICU status |  |  | 0.21 |
| yes | 80 (47.06) | 28 (43.75) |  |
| no | 90 (52.94) | 36 (56.25) |  |
| GCS score | 15 (11-15) | 14 (12, 15) | 0.02 |
| Injuries |  |  | 0.00 |
| Epidural | 17 (10.00) | 23 (35.94) |  |
| Subdural | 57 (33.53) | 13 (20.31) |  |
| Intracerebral | 96 (56.47) | 28 (43.75) |  |
| Brain surgery status |  |  | 0.27 |
| yes | 104 (61.18) | 34 (53.13) |  |
| no | 66 (38.82) | 30 (46.88) |  |
| Intracranial infection status |  |  | 0.29 |
| yes | 3 (1.76) | 0 (0.00) |  |
| no | 167 (98.24) | 64 (100.00) |  |
| Smoking status |  |  | 0.61 |
| yes | 51 (30.00) | 17 (26.56) |  |
| no | 119 (70.00) | 47 (73.44) |  |
| Alcohol consumption status |  |  | 0.64 |
| yes | 31 (18.24) | 10 (15.63) |  |
| no | 139 (81.76) | 54 (84.38) |  |
| Cerebrospinal fluid leakage status |  |  | 0.02 |
| yes | 24 (14.12) | 2 (3.13) |  |
| no | 146 (85.88) | 62 (96.88) |  |
| Skull fracture status |  |  | 0.25 |
| yes | 76 (44.71) | 34 (53.13) |  |
| no | 94 (55.29) | 30 (46.88) |  |
| Temperature on admission, median (IQR), °C | 36.5 (36.1-36.8) | 36.4 (36.0-36.925) | 0.75 |
| Temperature at discharge, median (IQR), °C | 36.4 (36.13-36.8) | 36.55 (36.3-36.8) | 0.09 |
| BMI^b^, mean (SD) | 22.80 (2.41) | 23.22 (2.59) | 0.25 |
| Mean arterial pressure on admission, mean (SD), mmHg | 99.46 (14.39) | 95.61 (13.44) | 0.06 |
| Mean arterial pressure at discharge, mean (SD), mmHg | 91.31 (10.54) | 87.59 (9.99) | 0.02 |
| Haemoglobin on admission, mean (SD), g/L | 126.38 (18.50) | 133.26 (17.71) | 0.01 |
| MCHC on admission, mean (SD), g/L | 337.58 (11.67) | 336.42 (9.48) | 0.48 |
| Platelet count on admission, mean (SD),109/L | 187.94 (61.03) | 195.38 (49.12) | 0.38 |
| NLR^c^ on admission, mean (SD), % | 11.01 (12.41) | 11.76 (8.00) | 0.26 |
| Haemoglobin at discharge, mean (SD), g/L | 122.82 (18.42) | 131.84 (22.90) | 0.00 |
| MCHC at discharge, mean (SD), g/L | 333.99 (20.25) | 336.14 (10.17) | 0.42 |
| NLR^c^ at discharge, mean (SD), % | 4.76 (3.71) | 4.22 (3.59) | 0.32 |
| Platelet count at discharge, mean (SD), 109/L | 228.50 (80.38) | 238.14 (87.45) | 0.43 |
| Albumin, mean (SD), g/L | 35.93 (5.26) | 37.64 (5.90) | 0.03 |
| γ-GT, mean (SD), U/L | 39.31 (35.09) | 33.70 (31.68) | 0.27 |
| Cholesterol, mean (SD), mmol/L | 4.06 (0.93) | 3.95 (0.74) | 0.39 |
| Triglyceride, mean (SD), mmol/L | 1.62 (0.93) | 1.83 (2.42) | 0.18 |
| HDL, mean (SD), mmol/L | 1.21 (0.32) | 1.44 (0.89) | 0.58 |
| LDL, mean (SD), mmol/L | 1.97 (0.58) | 1.91 (0.49) | 0.47 |
| Creatinine, mean (SD), μmol/L | 79.06 (19.14) | 95.68 (178.04) | 0.23 |
| Serum potassium, mean (SD), mmol/L | 4.28 (0.38) | 4.33 (0.40) | 0.41 |
| Blood glucose, mean (SD), mmol/L | 6.25 (2.13) | 5.80 (1.65) | 0.13 |
| CRP, mean (SD), mg/L | 13.53 (15.39) | 11.20 (13.87) | 0.29 |
| D-dimer, mean (SD), mg/L | 3.53 (6.02) | 3.58 (7.30) | 0.96 |
| Marshall score |  |  | 0.01 |
| 1 | 1 (0.59) | 0 (0.11) |  |
| 2 | 21 (12.35) | 12 (18.75) |  |
| 3 | 31 (18.24) | 21 (32.81) |  |
| 4 | 5 (2.94) | 0 (0.00) |  |
| 5 | 14 (8.24) | 5 (6.25) |  |
| 6 | 98 (57.65) | 27 (42.19) |  |
| Helsinki score, mean (SD) | 4.68 (2.83) | 1.19 (2.46) | 0.00 |
|  |  |  |  |

Abbreviations: NICU, neurological intensive care unit; GCS score, Glasgow coma scale score; BMI, body mass index; MCHC, mean corpuscular haemoglobin concentration; NLR, neutrophil-to-lymphocyte ratio; γ-GT, glutamyl transpeptidase; HDL, high-density lipoprotein; LDL, low-density lipoprotein; CRP, C-reactive protein. On admission means within 24 hours of admission; at discharge means within 24 hours before discharge.

^a^Data are presented as the number (percentage) of patients unless otherwise indicated.

^b^ Body mass index is calculated as weight in kilograms divided by height in metres squared.

^c^ The neutrophil-to-lymphocyte ratio is calculated as the neutrophil count divided by the lymphocyte count.

| **Table S3. Net benefits for different threshold probabilities in the Training cohort** | | | |
| --- | --- | --- | --- |
| Threshold | All | None | Predictor |
| 0.01 | 0.72373306 | 0 | 0.72373306 |
| 0.02 | 0.72091401 | 0 | 0.72091401 |
| 0.03 | 0.71803683 | 0 | 0.71803683 |
| 0.04 | 0.71509972 | 0 | 0.71509972 |
| 0.05 | 0.71210076 | 0 | 0.71210076 |
| 0.06 | 0.70903801 | 0 | 0.70985634 |
| 0.07 | 0.70590938 | 0 | 0.70880434 |
| 0.08 | 0.70271275 | 0 | 0.70680045 |
| 0.09 | 0.69944585 | 0 | 0.70108951 |
| 0.10 | 0.69610636 | 0 | 0.70037987 |
| 0.11 | 0.69269183 | 0 | 0.68990685 |
| 0.12 | 0.68919969 | 0 | 0.68861694 |
| 0.13 | 0.68562727 | 0 | 0.68621672 |
| 0.14 | 0.68197177 | 0 | 0.68018287 |
| 0.15 | 0.67823027 | 0 | 0.67998994 |
| 0.16 | 0.67439967 | 0 | 0.67846968 |
| 0.17 | 0.67047678 | 0 | 0.67789105 |
| 0.18 | 0.66645820 | 0 | 0.67656869 |
| 0.19 | 0.66234040 | 0 | 0.67632162 |
| 0.20 | 0.65811966 | 0 | 0.67521368 |
| 0.21 | 0.65379206 | 0 | 0.66877637 |
| 0.22 | 0.64935350 | 0 | 0.66228359 |
| 0.23 | 0.64479964 | 0 | 0.66128316 |
| 0.24 | 0.64012596 | 0 | 0.66171840 |
| 0.25 | 0.63532764 | 0 | 0.65954416 |
| 0.26 | 0.63039963 | 0 | 0.65881266 |
| 0.27 | 0.62533661 | 0 | 0.65659759 |
| 0.28 | 0.62013295 | 0 | 0.65432099 |
| 0.29 | 0.61478271 | 0 | 0.65198026 |
| 0.30 | 0.60927961 | 0 | 0.64957265 |
| 0.31 | 0.60361699 | 0 | 0.64709526 |
| 0.32 | 0.59778783 | 0 | 0.64454500 |
| 0.33 | 0.59178467 | 0 | 0.64191861 |
| 0.34 | 0.58559959 | 0 | 0.63921264 |
| 0.35 | 0.57922419 | 0 | 0.63642341 |
| 0.36 | 0.57264957 | 0 | 0.63354701 |
| 0.37 | 0.56586623 | 0 | 0.62630579 |
| 0.38 | 0.55886407 | 0 | 0.62586159 |
| 0.39 | 0.55163234 | 0 | 0.62554295 |
| 0.40 | 0.54415954 | 0 | 0.62250712 |
| 0.41 | 0.53643343 | 0 | 0.61936839 |
| 0.42 | 0.52844091 | 0 | 0.61612143 |
| 0.43 | 0.52016794 | 0 | 0.61171090 |
| 0.44 | 0.51159951 | 0 | 0.60836386 |
| 0.45 | 0.50271950 | 0 | 0.60062160 |
| 0.46 | 0.49351060 | 0 | 0.59702437 |
| 0.47 | 0.48395420 | 0 | 0.59329140 |
| 0.48 | 0.47403024 | 0 | 0.58941486 |
| 0.49 | 0.46371711 | 0 | 0.58538629 |
| 0.50 | 0.45299145 | 0 | 0.57264957 |
| 0.51 | 0.44182801 | 0 | 0.56828885 |
| 0.52 | 0.43019943 | 0 | 0.56374644 |
| 0.53 | 0.41807601 | 0 | 0.56382979 |
| 0.54 | 0.40542549 | 0 | 0.55908584 |
| 0.55 | 0.39221273 | 0 | 0.55508072 |
| 0.56 | 0.37839938 | 0 | 0.54584305 |
| 0.57 | 0.36394355 | 0 | 0.54631286 |
| 0.58 | 0.34879935 | 0 | 0.54110704 |
| 0.59 | 0.33291641 | 0 | 0.53564728 |
| 0.60 | 0.31623932 | 0 | 0.52991453 |
| 0.61 | 0.29870699 | 0 | 0.52388779 |
| 0.62 | 0.28025191 | 0 | 0.52024291 |
| 0.63 | 0.26079926 | 0 | 0.50958651 |
| 0.64 | 0.24026591 | 0 | 0.49857550 |
| 0.65 | 0.21855922 | 0 | 0.48717949 |
| 0.66 | 0.19557567 | 0 | 0.48366013 |
| 0.67 | 0.17119917 | 0 | 0.47604248 |
| 0.68 | 0.14529915 | 0 | 0.48611111 |
| 0.69 | 0.11772815 | 0 | 0.47835677 |
| 0.70 | 0.08831909 | 0 | 0.46581197 |
| 0.71 | 0.05688182 | 0 | 0.45269673 |
| 0.72 | 0.02319902 | 0 | 0.44322344 |
| 0.73 | -0.01297879 | 0 | 0.42877493 |
| 0.74 | -0.05193951 | 0 | 0.41715976 |
| 0.75 | -0.09401709 | 0 | 0.41880342 |
| 0.76 | -0.13960114 | 0 | 0.43019943 |
| 0.77 | -0.18914902 | 0 | 0.41081382 |
| 0.78 | -0.24320124 | 0 | 0.42074592 |
| 0.79 | -0.30240130 | 0 | 0.40537241 |
| 0.80 | -0.36752137 | 0 | 0.40598291 |
| 0.81 | -0.43949618 | 0 | 0.38079172 |
| 0.82 | -0.51946819 | 0 | 0.36277303 |
| 0.83 | -0.60884867 | 0 | 0.35545500 |
| 0.84 | -0.70940171 | 0 | 0.32264957 |
| 0.85 | -0.82336182 | 0 | 0.32478632 |
| 0.86 | -0.95360195 | 0 | 0.29365079 |
| 0.87 | -1.10387903 | 0 | 0.34122288 |
| 0.88 | -1.27920228 | 0 | 0.33903134 |
| 0.89 | -1.48640249 | 0 | 0.31002331 |
| 0.90 | -1.73504274 | 0 | 0.31623932 |
| 0.91 | -2.03893637 | 0 | 0.25878443 |
| 0.92 | -2.41880342 | 0 | 0.30769231 |
| 0.93 | -2.90720391 | 0 | 0.36324786 |
| 0.94 | -3.55840456 | 0 | 0.27777778 |
| 0.95 | -4.47008547 | 0 | 0.05555556 |
| 0.96 | -5.83760684 | 0 | 0.00000000 |
| 0.97 | -8.11680912 | 0 | 0.00000000 |
| 0.98 | -12.67521368 | 0 | 0.00000000 |
| 0.99 | -26.35042735 | 0 | 0.00000000 |

| **Table S4. Net benefits for different threshold probabilities in the validation cohort** | | | |
| --- | --- | --- | --- |
| **Threshold** | **All** | **None** | **Predictor** |
| 0.01 | 0.59114959 | 0 | 0.59114959 |
| 0.02 | 0.58697765 | 0 | 0.58697765 |
| 0.03 | 0.58271969 | 0 | 0.58271969 |
| 0.04 | 0.57837302 | 0 | 0.57837302 |
| 0.05 | 0.57393484 | 0 | 0.57393484 |
| 0.06 | 0.56940223 | 0 | 0.56940223 |
| 0.07 | 0.56477215 | 0 | 0.56746032 |
| 0.08 | 0.56004141 | 0 | 0.56728778 |
| 0.09 | 0.55520670 | 0 | 0.55272109 |
| 0.10 | 0.55026455 | 0 | 0.55158730 |
| 0.11 | 0.54521134 | 0 | 0.55096308 |
| 0.12 | 0.54004329 | 0 | 0.54924242 |
| 0.13 | 0.53475643 | 0 | 0.54597701 |
| 0.14 | 0.52934662 | 0 | 0.54651163 |
| 0.15 | 0.52380952 | 0 | 0.54551821 |
| 0.16 | 0.51814059 | 0 | 0.54478458 |
| 0.17 | 0.51233505 | 0 | 0.54432014 |
| 0.18 | 0.50638792 | 0 | 0.52961672 |
| 0.19 | 0.50029394 | 0 | 0.52674897 |
| 0.20 | 0.49404762 | 0 | 0.52380952 |
| 0.21 | 0.48764316 | 0 | 0.52079566 |
| 0.22 | 0.48107448 | 0 | 0.52106227 |
| 0.23 | 0.47433519 | 0 | 0.51808905 |
| 0.24 | 0.46741855 | 0 | 0.51503759 |
| 0.25 | 0.46031746 | 0 | 0.51190476 |
| 0.26 | 0.45302445 | 0 | 0.50868726 |
| 0.27 | 0.44553164 | 0 | 0.49347684 |
| 0.28 | 0.43783069 | 0 | 0.49007937 |
| 0.29 | 0.42991281 | 0 | 0.48658618 |
| 0.30 | 0.42176871 | 0 | 0.48299320 |
| 0.31 | 0.41338854 | 0 | 0.47929607 |
| 0.32 | 0.40476190 | 0 | 0.47549020 |
| 0.33 | 0.39587775 | 0 | 0.47157072 |
| 0.34 | 0.38672439 | 0 | 0.45562771 |
| 0.35 | 0.37728938 | 0 | 0.43956044 |
| 0.36 | 0.36755952 | 0 | 0.43526786 |
| 0.37 | 0.35752079 | 0 | 0.43083900 |
| 0.38 | 0.34715822 | 0 | 0.42626728 |
| 0.39 | 0.33645589 | 0 | 0.42154567 |
| 0.40 | 0.32539683 | 0 | 0.42460317 |
| 0.41 | 0.31396287 | 0 | 0.41989508 |
| 0.42 | 0.30213465 | 0 | 0.41502463 |
| 0.43 | 0.28989140 | 0 | 0.41896408 |
| 0.44 | 0.27721088 | 0 | 0.41411565 |
| 0.45 | 0.26406926 | 0 | 0.38528139 |
| 0.46 | 0.25044092 | 0 | 0.38007055 |
| 0.47 | 0.23629829 | 0 | 0.38522013 |
| 0.48 | 0.22161172 | 0 | 0.39102564 |
| 0.49 | 0.20634921 | 0 | 0.38608777 |
| 0.50 | 0.19047619 | 0 | 0.40476190 |
| 0.51 | 0.17395530 | 0 | 0.40038873 |
| 0.52 | 0.15674603 | 0 | 0.39583333 |
| 0.53 | 0.13880446 | 0 | 0.39108409 |
| 0.54 | 0.12008282 | 0 | 0.38612836 |
| 0.55 | 0.10052910 | 0 | 0.39550265 |
| 0.56 | 0.08008658 | 0 | 0.39069264 |
| 0.57 | 0.05869324 | 0 | 0.37375415 |
| 0.58 | 0.03628118 | 0 | 0.36848073 |
| 0.59 | 0.01277584 | 0 | 0.36295006 |
| 0.60 | -0.01190476 | 0 | 0.37500000 |
| 0.61 | -0.03785104 | 0 | 0.36965812 |
| 0.62 | -0.06516291 | 0 | 0.35213033 |
| 0.63 | -0.09395109 | 0 | 0.34620335 |
| 0.64 | -0.12433862 | 0 | 0.33994709 |
| 0.65 | -0.15646259 | 0 | 0.33333333 |
| 0.66 | -0.19047619 | 0 | 0.34943978 |
| 0.67 | -0.22655123 | 0 | 0.33116883 |
| 0.68 | -0.26488095 | 0 | 0.32440476 |
| 0.69 | -0.30568356 | 0 | 0.31720430 |
| 0.70 | -0.34920635 | 0 | 0.30952381 |
| 0.71 | -0.39573071 | 0 | 0.28940887 |
| 0.72 | -0.44557823 | 0 | 0.31122449 |
| 0.73 | -0.49911817 | 0 | 0.29144621 |
| 0.74 | -0.55677656 | 0 | 0.27106227 |
| 0.75 | -0.61904762 | 0 | 0.26190476 |
| 0.76 | -0.68650794 | 0 | 0.24007937 |
| 0.77 | -0.75983437 | 0 | 0.21739130 |
| 0.78 | -0.83982684 | 0 | 0.18181818 |
| 0.79 | -0.92743764 | 0 | 0.13321995 |
| 0.80 | -1.02380952 | 0 | 0.10714286 |
| 0.81 | -1.13032581 | 0 | 0.09147870 |
| 0.82 | -1.24867725 | 0 | 0.18253968 |
| 0.83 | -1.38095238 | 0 | 0.15896359 |
| 0.84 | -1.52976190 | 0 | 0.19642857 |
| 0.85 | -1.69841270 | 0 | 0.18650794 |
| 0.86 | -1.89115646 | 0 | 0.24829932 |
| 0.87 | -2.11355311 | 0 | 0.21794872 |
| 0.88 | -2.37301587 | 0 | 0.19841270 |
| 0.89 | -2.67965368 | 0 | 0.15367965 |
| 0.90 | -3.04761905 | 0 | 0.11904762 |
| 0.91 | -3.49735450 | 0 | 0.02248677 |
| 0.92 | -4.05952381 | 0 | -0.04166667 |
| 0.93 | -4.78231293 | 0 | 0.04761905 |
| 0.94 | -5.74603175 | 0 | 0.00000000 |
| 0.95 | -7.09523810 | 0 | 0.00000000 |
| 0.96 | -9.11904762 | 0 | 0.00000000 |
| 0.97 | -12.49206349 | 0 | 0.00000000 |
| 0.98 | -19.23809524 | 0 | 0.00000000 |
| 0.99 | -39.47619048 | 0 | 0.00000000 |


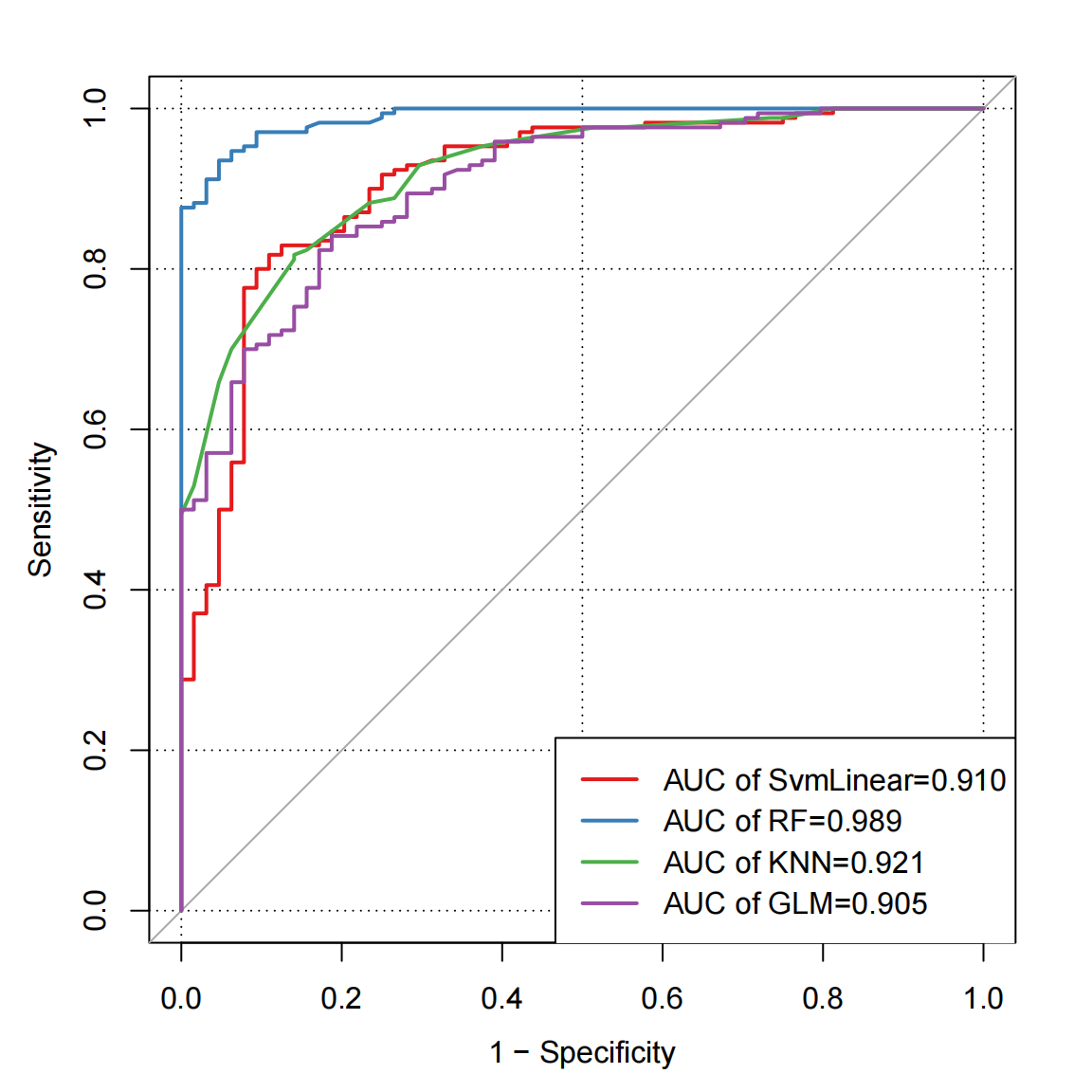


Figure S1A. ROC curve for cognitive impairment prediction in the training cohort

SVMLinear model AUC: 0.910, 95%CI: 0.866-0.953, sensitivity: 0.818, 95%CI: 0.760-0.876, specificity: 0.891, 95%CI: 0.814-0.967

RF model AUC: 0.989, 95%CI: 0.980-0.998, sensitivity: 0.935, 95%CI: 0.88-0.972, specificity: 0.953, 95%CI: 0.901-1.000.

KNN model AUC: 0.921, 95%CI: 0.8866-0.956, sensitivity: 0.818, 95%CI: 0.760-0.876, specificity: 0.859, 95%CI: 0.774-0.945.

GLM model AUC: 0.905, 95%CI: 0.866-0.944, sensitivity: 0.841, 95%CI: 0.7866-0.896, specificity: 0.812, 95%CI: 0.717-0.908.

Validation cohort (Figure 2. )


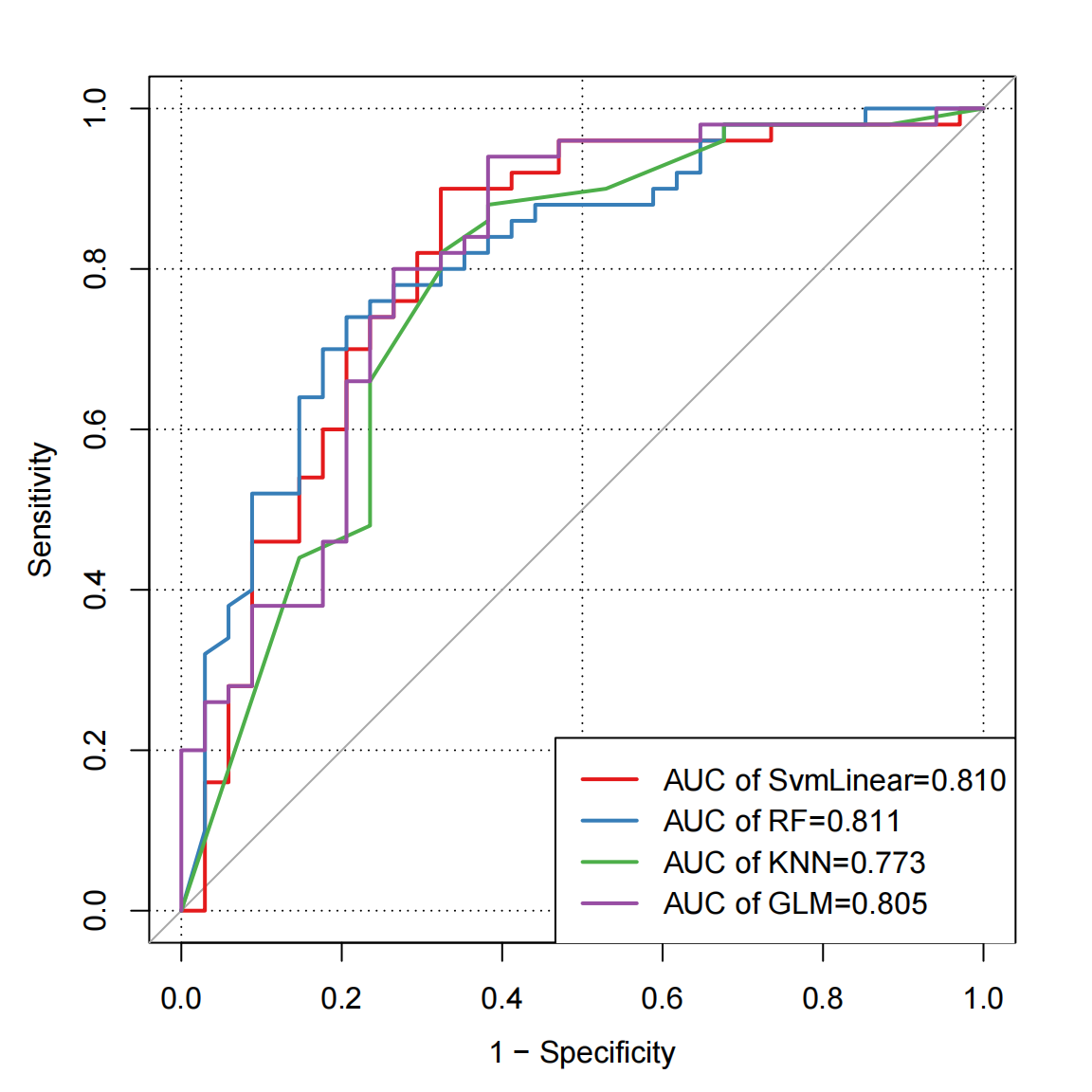


Figure S1B. ROC curve for cognitive impairment prediction in the validation cohort

SVMLinear model AUC: 0.810, 95%CI: 0.708-0.912, sensitivity: 0.900, 95%CI: 0.817-0.983, specificity: 0.676, 95%CI: 0.519-0.834.

RF model AUC: 0.811, 95%CI: 0.716-0.907, sensitivity: 0.740, 95%CI: 0.618-0.862, specificity: 0.794, 95%CI: 0.658-0.930.

KNN model AUC: 0.773, 95%CI: 0.666-0.880, sensitivity: 0.880, 95%CI: 0.790-0.970, specificity: 0.618, 95%CI: 0.454-0.781.

GLM model AUC: 0.805, 95%CI: 0.705-0.906, sensitivity: 0.940, 95%CI: 0.874-1.000, specificity: 0.618, 95%CI: 0.454-0.781.

1. Calibration degree evaluation(Figure 3-4. )


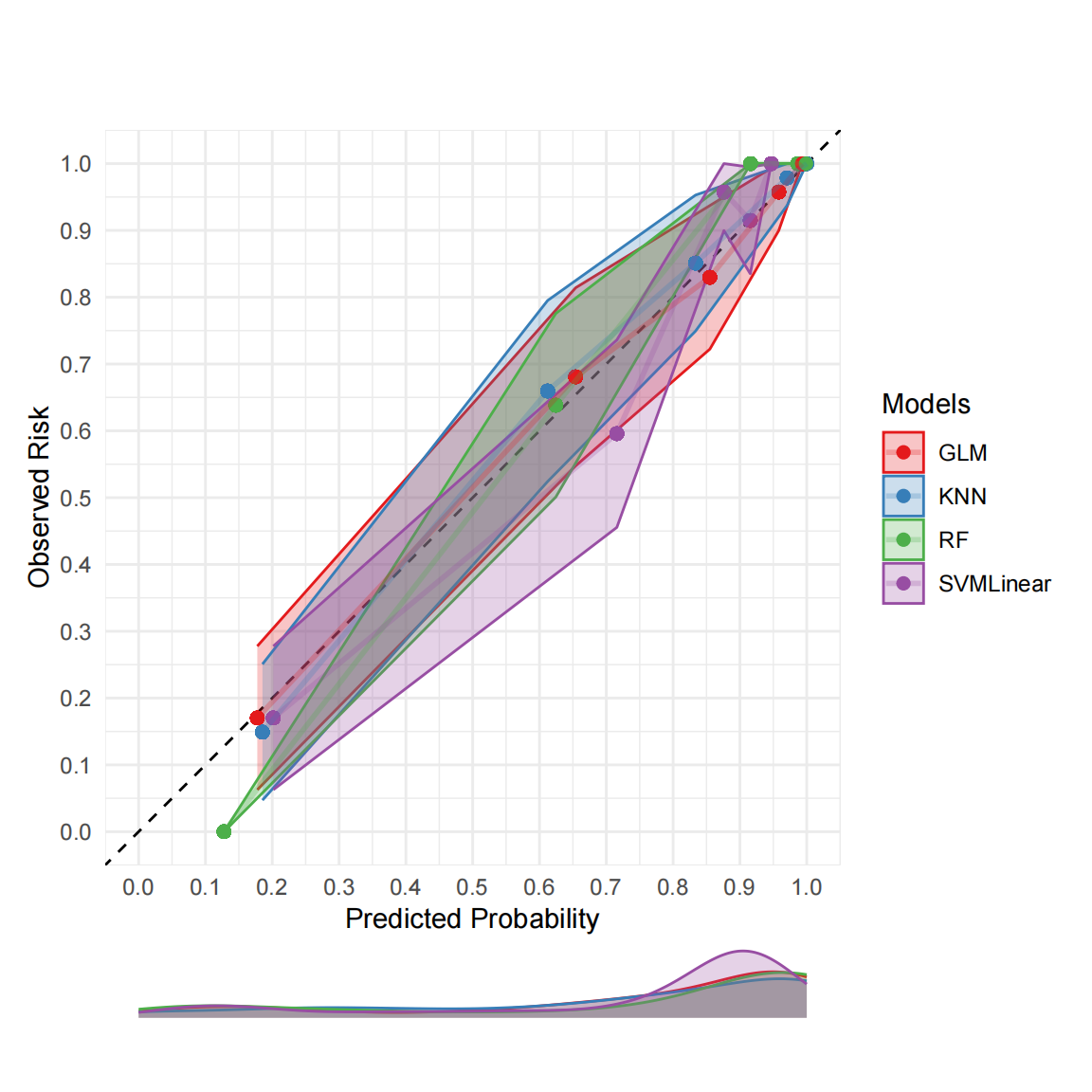


FigureS2A. Calibration curves for predicting cognitive impairment in the training cohorts


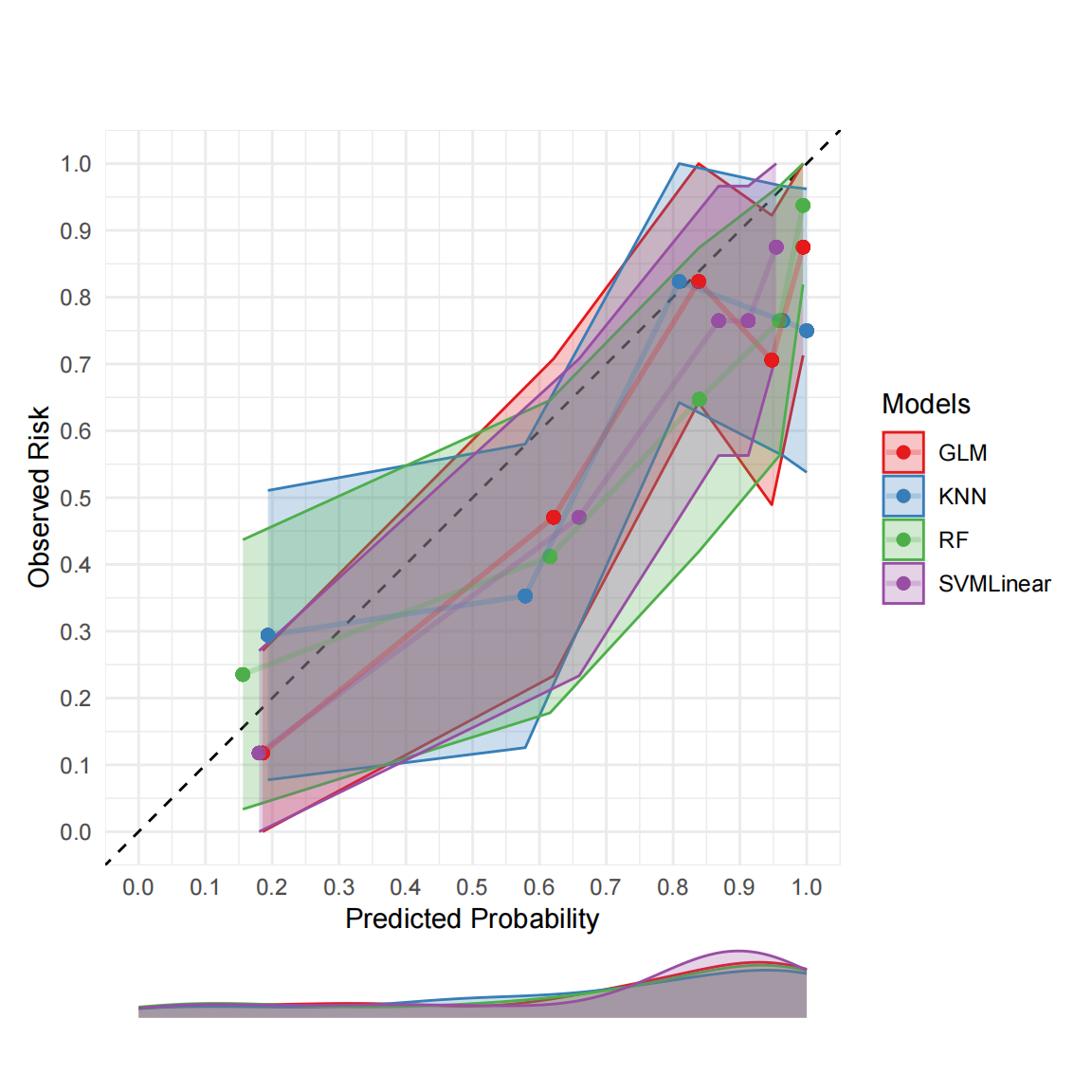


Figure S2B. Calibration curve for cognitive impairment prediction in the validation cohorts

1. Effectiveness evaluation(Figure 5-6. )


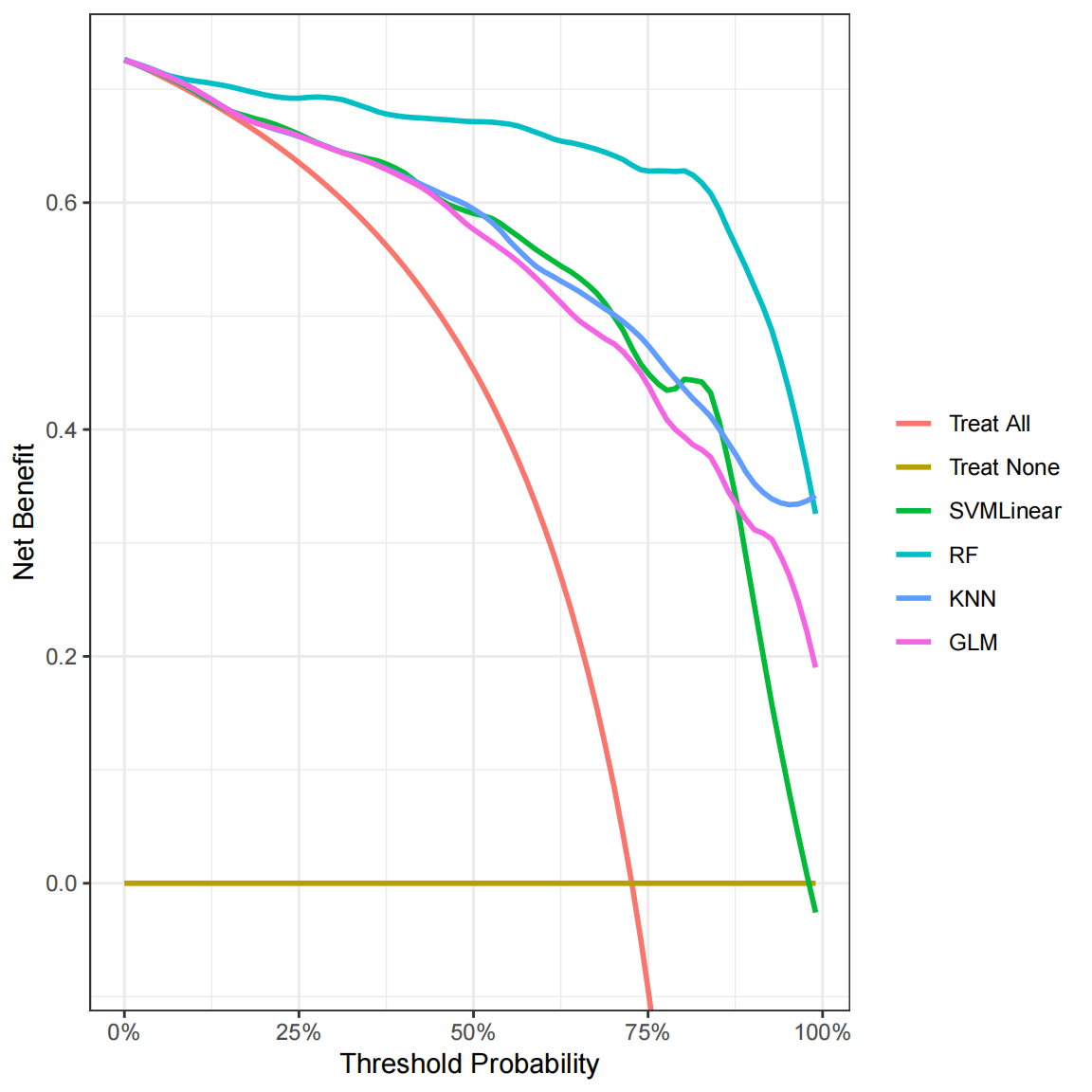


Figure S3A. Decision curve analysis for cognitive impairment pediction in the training cohorts


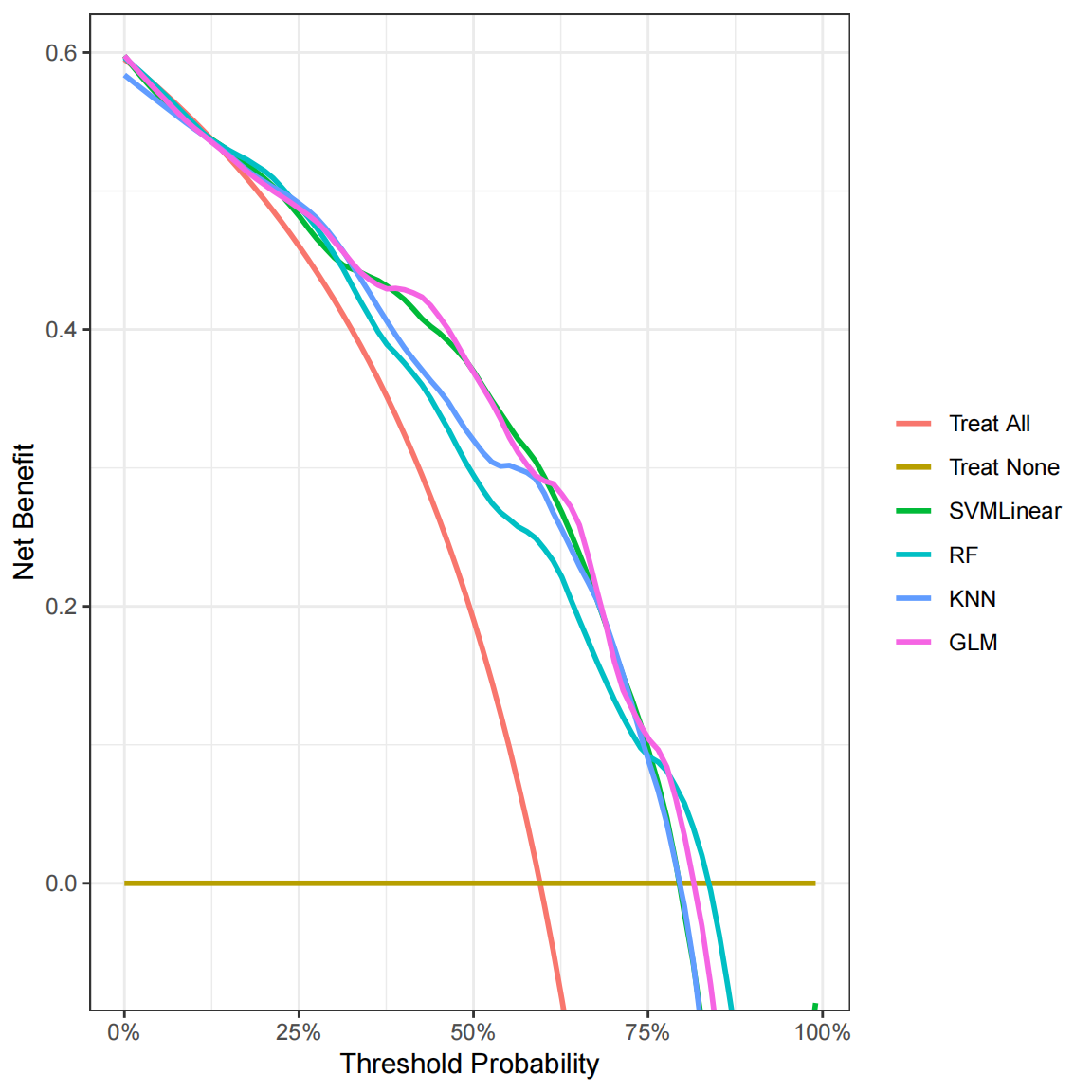


Figure S3B. Decision curve analysis for cognitive impairment prediction in the validation cohorts
